# A tale of two gradients: differences between the left and right hemispheres predict semantic cognition

**DOI:** 10.1007/s00429-021-02374-w

**Published:** 2021-09-12

**Authors:** Tirso Rene del Jesus Gonzalez Alam, Brontë L. A. Mckeown, Zhiyao Gao, Boris Bernhardt, Reinder Vos de Wael, Daniel S. Margulies, Jonathan Smallwood, Elizabeth Jefferies

**Affiliations:** 1 Department of Psychology, University of York, York, UK; 2 McConnell Brain Imaging Centre, Montreal Neurological Institute and Hospital, McGill University, Montreal, QC Canada; 3 Centre National de la Recherche Scientifique (CNRS) and Université de Paris, INCC UMR 8002, Paris, France; 4 Queen’s University, Kingston, ON Canada

**Keywords:** Gradients, Intrinsic connectivity, Semantic cognition, Hemispheric specialization, Laterality, fMRI

## Abstract

Decomposition of whole-brain functional connectivity patterns reveals a principal gradient that captures the separation of sensorimotor cortex from heteromodal regions in the default mode network (DMN). Functional homotopy is strongest in sensorimotor areas, and weakest in heteromodal cortices, suggesting there may be differences between the left and right hemispheres (LH/RH) in the principal gradient, especially towards its apex. This study characterised hemispheric differences in the position of large-scale cortical networks along the principal gradient, and their functional significance. We collected resting-state fMRI and semantic, working memory and non-verbal reasoning performance in 175 + healthy volunteers. We then extracted the principal gradient of connectivity for each participant, tested which networks showed significant hemispheric differences on the gradient, and regressed participants’ behavioural efficiency in tasks outside the scanner against interhemispheric gradient differences for each network. LH showed a higher overall principal gradient value, consistent with its role in heteromodal semantic cognition. One frontotemporal control subnetwork was linked to individual differences in semantic cognition: when it was nearer heteromodal DMN on the principal gradient in LH, participants showed more efficient semantic retrieval—and this network also showed a strong hemispheric difference in response to semantic demands but not working memory load in a separate study. In contrast, when a dorsal attention subnetwork was closer to the heteromodal end of the principal gradient in RH, participants showed better visual reasoning. Lateralization of function may reflect differences in connectivity between control and heteromodal regions in LH, and attention and visual regions in RH.

**Supplementary Information:**

The online version contains supplementary material available at 10.1007/s00429-021-02374-w.

## Introduction

Contemporary accounts of brain organisation conceptualise cognition as reflecting interactions of large-scale networks of brain regions, organised in a systematic fashion along cortical gradients. These gradients capture similarities in connectivity patterns across disparate areas of the cortex (Bressler and Menon 2010; Margulies et al. 2016; Medaglia et al. 2015; Paquola et al. 2018; Yeo et al. 2011). Cortical gradients provide a new tool for understanding patterns of hemispheric specialisation, since networks with lateralised connectivity will occupy different positions along these gradients in the left (LH) and right hemispheres (RH). This study exploits the potential of cortical gradients to uncover hemispheric differences in patterns of intrinsic connectivity, (i) by assessing the position of canonical networks in the left and right hemisphere along gradients derived bilaterally, and (ii) by examining the functional significance of these hemispheric differences for the highly left-lateralised domain of semantic cognition, compared with other cognitive domains (working memory (WM) and visual reasoning) that are expected to show different patterns of lateralisation.

The principal gradient, which explains the most variance in whole-brain decompositions of intrinsic connectivity, captures the separation between sensory-motor cortex and heteromodal Default Mode Network (DMN) (Huntenburg et al. 2018; Margulies et al. 2016). In this way, it relates to previously described cortical hierarchies that extract progressively more complex or heteromodal information from sensory inputs, or that maintain more abstract goals for action, in lateral and medial temporal lobes, and lateral and medial prefrontal cortex (Badre 2008; Badre and D’Esposito 2007; Bajada et al. 2017, 2019; Fuster 2001; Jackson et al. 2017, 2019; Koechlin et al. 2003; Petrides 2005; Thiebaut De Schotten et al. 2017). The principal gradient goes beyond these observations to explain why similar hierarchies occur in multiple brain regions. The principal gradient is correlated with physical distance along the cortical surface from primary systems, with the DMN falling at a maximum distance from sensory and motor systems in multiple locations across the cortex. Since DMN is a highly distributed network, with multiple nodes located in distant brain regions, the functional transitions captured by the principal gradient are repeated across the cortex, and these are seen in both hemispheres. The principal gradient also captures the sequence of networks found along the cortical surface—from DMN, through frontoparietal control networks, to attention networks (Dorsal and Ventral, DAN and VAN) and finally primary somatomotor and visual networks. A recent study showed that when gradient decomposition is performed for the two hemispheres separately, both hemispheres contain a similar (but not identical) principal gradient (Liang et al. 2021). However, the functional relevance of these similarities and differences between the left and right hemisphere has not been established.

Patterns of intrinsic connectivity tend to be highly symmetrical, with the strongest time-series correlations seen between homotopic regions that occupy the same position in the two hemispheres (Jo et al. 2012). However, symmetrical patterns of connectivity are weaker within heteromodal networks towards the DMN apex of the gradient (Raemaekers et al. 2018). These increasing asymmetries are related to structural connectivity: primary cortices are connected across the hemispheres through fast fibres of the corpus callosum, while heteromodal cortices are connected by slower fibres that show less homotopic connectivity (Stark et al. 2008). A recent study using large-scale novel meta-analytic and voxel mirroring methods confirmed that areas with less similar connectivity across hemispheres are associated with heteromodal functions, such as memory, language and executive control (Mancuso et al. 2019). Moreover, higher-order networks, including DMN, frontoparietal network (FPN) and dorsal attention network (DAN), show the highest degrees of interhemispheric differences in intrinsic connectivity (Karolis et al. 2019; Wang et al. 2014). These lateralised patterns of connectivity have functional significance, giving rise to lateralised functions like verbal semantics and other components of language (Joliot et al. 2016; Knecht et al. 2000) and aspects of attention (Bartolomeo and Seidel Malkinson 2019). For example, Gotts et al. (2013) identified that a ‘segregation’ mode of lateralisation in the left hemisphere (i.e., heightened intrinsic connectivity with other left hemisphere regions), conferred behavioural advantages in a verbal semantic task (vocabulary). In contrast, cross-hemisphere connections for the right hemisphere were related to better visual reasoning (block design). Given that segregated connectivity is also associated with higher-order heteromodal networks, we would expect this left hemisphere semantic pattern to involve lateralised connectivity at the heteromodal end of the gradient.

Previous studies have identified hemispheric differences in control networks, situated between DMN and sensory-motor cortex. In the left hemisphere, the frontoparietal control network couples preferentially to DMN and language regions, while in the right hemisphere, this network shows stronger connectivity to attentional regions (Wang et al. 2014). These findings suggest that control networks might be critical for the emergence of lateralised cognition. In line with this view, the most lateralised regions of the semantic network are associated with controlled semantic retrieval, as opposed to conceptual representation (Gonzalez Alam et al. 2019). Furthermore, the clustering of connectivity patterns within the FPN across hemispheres reveals a bipartite organisation, with one subnetwork showing more intrinsic connectivity to DMN, whilst the other shows more connectivity to DAN (Dixon et al. 2018). These subnetworks may support the capacity of the FPN to couple efficiently with the DAN and DMN, depending on the task (Niendam et al. 2012; Spreng et al. 2013; Vincent et al. 2008; Wang et al. 2014). These observations collectively give rise to the hypothesis that differences in network interactions between the hemispheres might be reflected in the location of control networks on the principal gradient, with left hemisphere control regions nearer to DMN, and right hemisphere control areas nearer to the sensory-motor end of the gradient. In line with this view, Davey et al. (2016) suggested that left-lateralised semantic control processes reflect an interaction of heteromodal conceptual representations, associated with DMN, and control processes that can promote the retrieval of currently-relevant aspects of knowledge, even when these are not dominant in long-term memory. Semantic cognition may be left lateralised because these DMN and control networks interact more strongly in the left hemisphere.

This study contrasted patterns of lateralisation for semantic cognition with working memory (Studies 1 and 2) and visual reasoning using matrix problems (Study 1). Since semantic cognition is thought to draw on left-lateralised interactions between DMN and control regions (Davey et al. 2016), working memory tasks provide an interesting contrast: increased working memory demands are expected to increase reliance on a bilateral multiple-demand network that supports executive demands across tasks (Duncan 2001, 2010; Fedorenko et al. 2013; Hugdahl et al. 2015), with the differential engagement of left and right hemispheres when verbal and spatial working memory tasks are compared (Emch et al. 2019; Hong et al. 2000). The multiple-demand network is adjacent to but somewhat spatially distinct from the semantic control network (Davey et al. 2016; Gao et al. 2021; Jackson 2021), and its recruitment typically shows less lateralisation (Camilleri et al. 2018; Müller et al. 2015; Rottschy et al. 2012). Visual reasoning tasks are also expected to show a distinct pattern of lateralisation compared with semantic cognition. While semantic cognition is strongly left-lateralised, meta-analytic and patient evidence suggests a bilateral basis for a wide variety of reasoning tasks (Hobeika et al. 2016; Wertheim and Ragni 2018; Shin and Jeon 2021; Gläscher et al. 2010). Shin and Jeon (2021) found common bilateral activation in multiple demand cortex across inductive and deductive tasks, with stronger responses in the right hemisphere for more complex tasks. In some studies, matrix reasoning is more right-lateralised than analogical reasoning, consistent with this task’s greater visual and spatial demands (Hobeika et al. 2016; but see also Wertheim and Ragni 2018). Visual reasoning tasks are expected to involve an interaction of control/attention networks with visual regions (Hearne et al. 2017), without strong engagement of memory processes in DMN; this pattern of network interaction might be bilateral or stronger in the right hemisphere. Structural equation modelling has shown that while executive and perceptual attention both contribute to the RAPM, executive control plays a larger role (Schweizer and Moosbrugger 2004; Ren et al. 2012; 2013). Although simple visual attention is thought to be right-lateralised (Kinsbourne 1987; Fink et al. 2000, 2001), the RAPM gives rise to bilateral responses with some evidence pointing to a right-lateralised bias under certain conditions (Prabhakaran et al. 1997; Bishop et al. 2008).

Study 1 examined the organisation of the principal gradient across the left and right human cerebral hemispheres in participants who took part in a resting-state scan (*N *= 253) and behavioural tasks in a separate session (*N *= 175). We considered how individual differences in semantic cognition related to the position of large-scale networks on the principal gradient of intrinsic connectivity defined by Margulies et al. (2016), in the left versus right hemispheres, deriving a hemispheric difference gradient score per network for each participant. The semantic component that we examined was derived from a wide variety of semantic tasks, and is likely to reflect the capacity to access relevant conceptual knowledge in different contexts. We would expect this component to be left-lateralised as all elements of the semantic cognition network appear to show a left-hemisphere bias in meta-analytic evidence (the map for the term ‘semantic cognition’ from Neurosynth, for example, is highly left-lateralised), with the possible exception of the anterior temporal lobe (Rice et al. 2015a, b; Jackson et al. 2017; Gonzalez Alam et al. 2019). These effects for semantic judgements were compared with Raven’s Advanced Progressive Matrices (RAPM), a measure of non-verbal reasoning, and Digit Span, a measure of verbal working memory, allowing us to determine whether hemispheric differences on the principal gradient are related in distinct ways to left-lateralised semantic cognition (Gonzalez Alam et al. 2019; Jackson 2021; Noonan et al. 2013), compared with other types of demanding cognition. Using similar methods, Mckeown et al. (2020) found associations between individual differences in gradient values and patterns of spontaneous thought, suggesting that variation in gradient organisation is reflected in people’s cognition and experience.

Having established that individual differences in semantic cognition were associated with the magnitude of hemispheric differences on the principal gradient in a specific control network in Study 1, we examined how semantic and non-semantic task demands modulated activation within this lateralised control network in Study 2. We re-analysed fMRI data examining parametric manipulations of difficulty in semantic and verbal working memory tasks (Gao et al. 2021). Controlled semantic retrieval demands were varied by presenting word pairs that were strongly or more weakly associated: previous studies have shown greater recruitment of the left-lateralised semantic control network when participants are required to identify weak associations that are not dominant within the semantic store (Jackson 2021; Noonan et al. 2013). Semantic control demands were compared with the effects of working memory load, since higher working memory demands increase the recruitment of bilateral multiple-demand cortex that is partially distinct from the semantic control network (Fedorenko et al. 2013). In this way, we assessed whether hemispheric differences in the position of large-scale networks on the principal gradient of intrinsic connectivity at rest corresponded with hemispheric differences in the recruitment of these networks during task performance.

## Methods

### Study 1

#### Participants

Two hundred and seventy-seven healthy participants were recruited from the University of York. Written informed consent was obtained for all participants and the study was approved by the York Neuroimaging Centre Ethics Committee. The participants were right-handed, native English speakers with normal/corrected vision. None of them had a history of psychiatric or neurological illness, severe claustrophobia, drug use that could alter cognitive functioning, or pregnancy. Twenty-four participants were excluded from fMRI analyses; two due to technical issues during the neuroimaging data acquisition, one due to a data processing error and twenty-one for excessive movement during the scan (Power et al. 2014; mean framewise displacement > 0.3 mm and/or more than 15% of their data affected by motion), resulting in a final cohort of *N *= 253 (169 females, mean ± SD age = 20.7 ± 2.4 years). A subset of 175 of these participants also completed a semantic relatedness judgement task, a digit span task and Raven’s Progressive Matrices (along with other behavioural tasks outside the scope of this study), in a separate session. While the current analysis of hemispheric gradient differences is novel, this data has been used in previous studies to examine the neural basis of memory and mind-wandering, including region-of-interest based connectivity analysis and cortical thickness investigations (Evans et al. 2020; Gonzalez Alam et al. 2018, 2019, 2021; Karapanagiotidis et al. 2017; Poerio et al. 2017; Sormaz et al. 2018; Turnbull et al. 2018; Vatansever et al. 2017; Wang et al. 2018a, b).

#### Procedure

All participants underwent a 9 min resting-state fMRI scan. During the scan, they were instructed to passively view a fixation cross and not to think of anything in particular. Immediately following the scan, they completed a 25-item experience-sampling questionnaire while still in the scanner; these data have been reported in Karapanagiotidis et al. (2020) and Mckeown et al. (2020). In two subsequent sessions, participants completed a battery of semantic and other cognitive tasks. These sessions included personality and wellbeing scales, and measures of perception, episodic memory, cognitive control and mind-wandering, which were beyond the scope of this investigation.

#### Materials

Given our focus is on the lateralisation of semantic cognition, we selected a semantic judgement test battery for analysis. We contrasted the pattern for left-lateralised semantic cognition with two non-semantic tasks expected to have a different pattern of lateralisation – Raven’s progressive matrices (Raven et al. 1994), involving visual reasoning, and forwards digit span adapted from the Weschler Adult Intelligence Scale (WAIS; Wechsler 1955). Raven’s progressive matrices engages bilateral visual and attentional control processes that have a right-hemisphere bias (Bishop et al. 2008; Corbetta et al. 2008; Haier et al. 1988; Prabhakaran et al. 1997; Ren et al. 2012; Schweizer and Moosbrugger 2004), while the digit span task engages left as well as right-lateralised aspects of multiple-demand cortex (Fedorenko et al. 2013; Hugdahl et al. 2015). This task comparison can therefore establish whether any hemispheric differences in gradient values relating to semantic cognition are specific to the semantic domain, or are found more generally for demanding tasks.

##### Semantic task

**Fig. 1:**
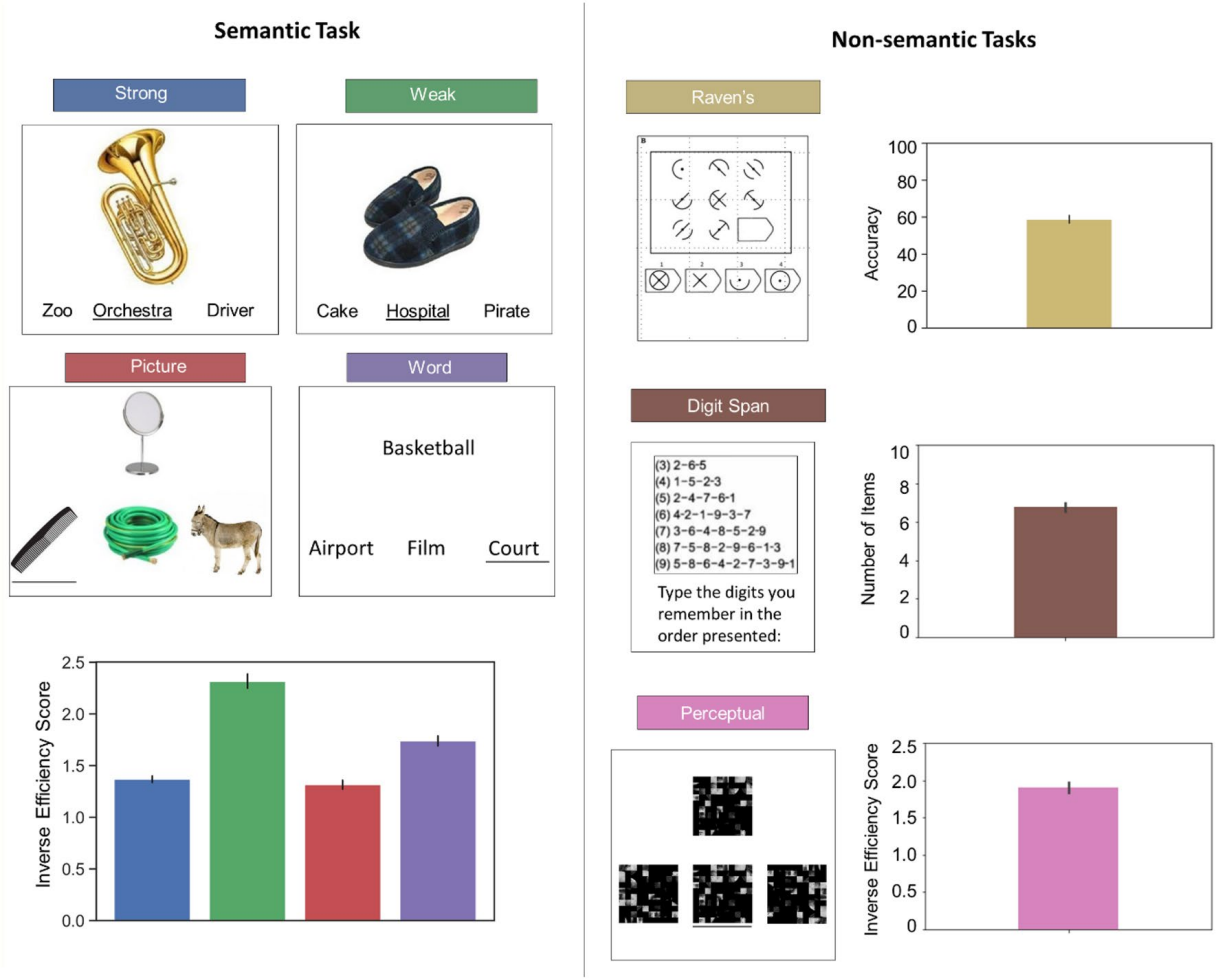
Illustration of the semantic (left panel) and non-semantic (right panel) tasks employed in this study. The bar plots are colour coded to match the examples depicting each condition of the tasks (i.e., the blue bar in the left panel corresponds to the ‘strong’ condition). Performance in the semantic and perceptual tasks are depicted here using inverse efficiency scores, in which larger numbers correspond to worse performance. Elsewhere, efficiency scores were multiplied by − 1, for interpretability. Digit span is expressed as a maximum number of items recalled, and Raven’s as accuracy (number of problems solved out of a maximum of 36). Error bars depict 95% confidence intervals

Participants performed semantic relatedness judgements that manipulated modality (words/pictures) and strength of association (weak/strong associates; see Fig. 1). The task employed a three-alternative forced-choice design: participants matched a probe stimulus on the screen with one of three possible targets, and pressed buttons to indicate their choice. Each trial consisted of a centrally presented probe preceded by a target and two unrelated distractors, which were targets in other trials. Trials started with a blank screen for 500 ms. The three response options were subsequently presented at the bottom of the screen for 900 ms (aligned horizontally, with the target in each location equally often). Finally, the probe was presented at the top of the screen. The probe and choices remained visible until the participant responded, or for a maximum of 3 s. Both response time (RT) and accuracy were recorded, and an efficiency score was calculated for each participant in each condition by dividing response times by accuracy, and multiplying that ratio by − 1, so that higher scores reflected better performance. This approach allowed us to control for speed-accuracy trade-offs when assessing associations with intrinsic connectivity and avoids the inflation of Type 1 errors that would result from running parallel analyses for accuracy and RT separately. The Supplementary Materials describe assessments of the suitability of the data for response efficiency analysis (see Supplementary Analysis: assumption check for efficiency scores).

The stimuli employed in the tasks were selected from a larger set of words and photographs used in previous experiments (Davey et al. 2015; Krieger-Redwood et al. 2015). The pictures were coloured photographs collected from the internet and re-sized to fit the trial structure (200 pixels, 72 dpi). All the coloured pictures and words were rated for familiarity using 7-point Likert scales, and imageability (> 500) from the MRC psycholinguistic database (Coltheart 1981; Wilson 1988). Lexical frequency for the words was obtained by the SUBTLEX-UK database (van Heuven et al. 2014) to allow matching on psycholinguistic properties. The strength of association between probe-target pairs was assessed using a 7-point Likert scale and differed significantly between conditions. There were no differences between strong and weak associations in word length, familiarity, imageability or lexical frequency. The order of trials within the blocks (conditions) was randomized across subjects. Each block contained 60 trials. The presentation of the blocks was interleaved.

##### Semantic dimensionality reduction

 Given that efficiency scores were correlated across the conditions of the task, we performed data-driven dimensionality reduction, which revealed a single semantic factor in the relatedness judgement task. PCA with varimax rotation yielded one single factor with Kaiser’s criterion above 1, explaining 75% of the variance (see Supplementary Fig. 9). Each participant’s efficiency scores in the four tasks were therefore summarised using a single score reflecting the single factor loading, which was carried forward into regression analysis after *z*-scoring and imputing any outlier above ± 2.5 with the mean. This metric is likely to reflect individual differences in general semantic performance, since it loaded on both verbal and pictorial semantic tasks, and conditions requiring little semantic control (i.e., strong associations) as well as greater semantic control (i.e., weak associations). The component scores are likely to reflect the global efficiency of conceptual retrieval resulting from both the strength of heteromodal semantic representations as well as the capacity to recruit control processes to support semantic cognition when needed. Summary measures of the behavioural data in each condition of the semantic task can be consulted in Supplementary Materials (Tables 4 and 5).

##### Raven’s advanced progressive matrices

The Ravens Advanced Progressive Matrices (Raven et al. 1994) is a measure of non-verbal reasoning that requires participants to identify meaningless visual patterns. The progressive matrices task included 36 questions, preceded by two practice trials. During the practice phase, participants were given feedback and task training, with no feedback for the reminder of the trials. For each problem, a set of 9 tiles (in a 3 × 3 design) were shown on the screen. All but one tile contained a pattern. At the bottom of the screen were 4 additional patterned tiles. Participants were required to select which tile would complete the pattern (see Fig. 1). Participants were given 20 min to complete as many problems as they could, and the problems got progressively more difficult.

##### Digit span

For the Digit Span Task, we used the stimuli and the scoring procedure described in the WAIS battery (Wechsler 1955), IV edition. For each trial, audio files of each digit were played in the sequential order reported in the WAIS battery. The instructions were presented at the beginning of each block asking participants to listen to the sequence of numbers and type them in the same order (see Fig. 1).

##### Perceptual figure matching task

The semantic, working memory and visual reasoning tasks used in this study varied in the number of response options, time available to respond and layout of presentation. Therefore, we included a behavioural covariate in the regression analysis that captured performance on a non-semantic figure matching task that was matched to the semantic battery in terms of the number of perceptual inputs, decision-making format, and mode of response. In this figure matching task, participants decided whether two scrambled images were mirror images of each other. The stimuli were 60 pixelated and scrambled black-and-white photographs of faces (Krieger-Redwood et al. 2013). Participants were asked to select the target that was identical to the probe; the distracters were the same images rotated by 180° or 270°.The task was split in two blocks of 30 trials each (see Fig. 1).

#### Neuroimaging

The MRI data acquisition and pre-processing steps reported in this paper are identical to the steps reported in Karapanagiotidis et al. (2020), and the dimension reduction steps are identical to the ones reported in Mckeown et al. (2020), as reproduced in the sections below.

##### MRI data acquisition

MRI data were acquired on a GE 3T Signa Excite HDx MRI scanner, equipped with an eight-channel phase-array head coil at York Neuroimaging Centre, University of York. For each participant, we acquired a sagittal isotropic 3D fast spoiled gradient-recalled echo T1-weighted structural scan (TR = 7.8 ms, TE = minimum full, flip angle = 20°, matrix = 256 × 256, voxel size = 1.13 × 1.13 × 1 mm^3^, FOV = 289 × 289 mm^2^). Resting-state fMRI data based on blood oxygen level-dependent contrast images with fat saturation were acquired using a gradient single-shot echo-planar imaging sequence (TE = minimum full (≈ 19 ms), flip angle = 90°, matrix = 64 × 64, FOV = 192 × 192 mm^2^, voxel size = 3 × 3 × 3 mm^3^, TR = 3000 ms, 60 axial slices with no gap and slice thickness of 3 mm). Scan duration was 9 min which allowed us to collect 180 whole-brain volumes.

##### MRI data pre-processing

fMRI data pre-processing was performed using SPM12 (http://www.fil.ion.ucl.ac.uk/spm) and the CONN toolbox (v.18b) (https://www.nitrc.org/projects/conn) (Whitfield-Gabrieli and Nieto-Castanon 2012) implemented in Matlab (R2018a) (https://uk.mathworks.com/products/matlab). Pre-processing steps followed CONN’s default pipeline and included motion estimation and correction by volume realignment using a six-parameter rigid body transformation, slice-time correction, and simultaneous grey matter (GM), white matter and cerebrospinal fluid (CSF) segmentation and normalisation to MNI152 stereotactic space (2 mm isotropic) of both functional and structural data. Following pre-processing, the following potential confounders were statistically controlled for: 6 motion parameters calculated at the previous step and their 1st and 2nd order derivatives, volumes with excessive movement (motion greater than 0.5 mm and global signal changes larger than *z* = 3), linear drifts, and five principal components of the signal from white matter and CSF using the CompCor approach (Behzadi et al. 2007). Finally, data were band-pass filtered between 0.01 and 0.1 Hz. No global signal regression was performed.

##### Gradient analysis

We obtained each participant’s gradient values for the first principal gradient (Margulies et al. 2016) following the methods described in Mckeown et al. (2020). Following pre-processing, the functional time-series from 400 ROIs based on the Schaefer parcellation (Schaefer et al. 2018; Yeo et al. 2011) were extracted for each individual. A connectivity matrix was then calculated using Pearson correlation resulting in a 400 × 400 connectivity matrix for each participant. These individual connectivity matrices were subjected to a Fisher *Z* transform prior to averaging, and then used to calculate a group-averaged connectivity matrix. The BrainSpace Toolbox (Vos de Wael et al. 2020) was used to extract ten group-level gradients from the group-averaged connectivity matrix (dimension reduction technique = diffusion embedding, kernel = normalized angle, sparsity = 0.9). This study was primarily focussed on the first gradient, which has well-described functional associations relevant to previous lateralisation findings; however, we extracted ten gradients to maximize the degree of fit between the group-averaged gradients and the individual-level first gradient (this method is justified by the analysis in Supplementary Table S2, which shows a higher degree of fit with the canonical group-level gradients established by Margulies et al. (2016) when extracting ten gradients compared with three). The variance explained by each group-averaged gradient is provided in Supplementary Fig. 6.

The group-level gradient solutions were aligned using Procrustes rotation to a subsample of the HCP dataset (*N *= 217, 122 women, mean ± SD age = 28.5 ± 3.7 y; for further details about subject selection and the benefits of this gradient alignment step, see Vos De Wael et al. 2018). The Procrustes rotation improves correspondence between the canonical gradients described by Margulies et al. (2016) and the group-level gradient solutions by rotating, translating and optionally scaling the group-level matrix to achieve maximum similarity with the target matrix minimizing the sum of squared differences. Procrustes rotation was chosen, as opposed to joint embedding, as it preserves the overall shape of the gradients (Vos de Wael et al. 2020). The first three group-averaged gradients, with and without alignment to the HCP data, are shown in Supplementary Fig. 7. To demonstrate the benefits of this alignment step, we calculated the similarity using Spearman Rank correlation between the first five aligned and unaligned group-level gradients to the first five gradients reported in Margulies et al. (2016), which were calculated using 820 participants over an hour-long resting-state scan. Alignment improved the stability of the group-level gradient templates by maximising the comparability of the solutions to those from the existing literature (i.e., Margulies et al. 2016; see our Supplementary Table S3).

Using identical parameters, individual-level gradients were then calculated for each individual using their 400 × 400 connectivity matrix. These individual-level gradient maps were aligned to the group-level gradient maps using Procrustes rotation to improve the comparison between the group-level gradients and individual-level gradients (*N* iterations = 10). This analysis resulted in ten group-level gradients and ten individual-level gradients for each participant explaining maximal whole-brain connectivity variance in descending order. Procrustes rotation was also used to address the reordering of gradient components (since sometimes gradients at the participant level are not in the same order as the canonical gradients at the group level). As stated above, this report focuses on the principal gradient (with supplementary analyses for Gradient 2), since this gradient captures the sequence of large-scale networks on the cortical surface. To demonstrate the variability of individual-level gradients, Supplementary Fig. 8 shows the highest, lowest, and median similarity gradient maps for the principal gradient.

##### Hemispheric difference analysis

As a first step for our analysis of interest, we obtained group averages of the principal gradient for each of the 400 parcels per participant (top row of Fig. 3). Since these parcels do not necessarily share homotopes across hemispheres, for the hemispheric difference analyses we summarised these values by averaging, for each participant, the parcels corresponding to each of the 17 networks described by Yeo et al. (2011). We will refer to these two levels of analyses as ‘parcel level’ and ‘network level’, respectively.

Next, we examined hemispheric differences across the 17 Yeo Network parcellation. We normalised each parcel’s principal gradient value within each participant using a minimum–maximum normalisation (0–100) before computing their network’s average, such that networks toward the lower end of the principal gradient have values closer to 0, and networks towards DMN have values close to 100 (the middle row of Fig. 3 shows the group average per network; the organisation of these networks are depicted in the bottom row of Fig. 3). We tested for hemispheric differences in the global gradient value by averaging all gradient values across the 17 Yeo networks within each hemisphere separately for each participant in the sample and comparing these means using a paired *t* test (left hemisphere vs right hemisphere).

Our next step involved subtracting the average of each right hemisphere network from its homotope in the left hemisphere, for each participant (we *z*-scored the results to produce a group difference map highlighting the networks with the most extreme differences shown in Fig. 4). We then performed a two-way repeated-measures ANOVA, using Hemisphere and Network as between-subject factors, to test for hemispheric differences at the network level. Having obtained significant main effects and an interaction, we conducted post-hoc non-parametric permutation testing with 5000 bootstrapped samples to compute the probability of obtaining a difference of gradient means across hemispheres as extreme as that empirically observed for each network by chance (Fig. 5). The non-parametric *p* values of these post-hoc tests were Bonferroni-corrected at an alpha = 0.05 for 17 multiple comparisons to guard against Type 1 errors. We only included those networks that showed significant hemispheric differences in the subsequent analyses.

##### Behavioural regressions

To examine whether hemispheric differences on the principal gradient across networks had behavioural consequences, we performed regression analyses relating participants’ performance outside the scanner on semantic and non-semantic tasks (working memory and visual reasoning) to the difference in principal gradient values across the hemispheres for each significant network. We entered each participant’s semantic factor loading as an Explanatory Variable (EV) into an Ordinary Least Squares (OLS) regression, using hemispheric difference scores on the principal gradient for each network as the dependent variable. The semantic factor was added together with participants’ *z*-scored performance on Raven’s matrices, digit span and perceptual judgements matched superficially to the semantic tasks. All four of these task EVs were entered together for each regression model. An additional analysis examined individual differences in variation between the conditions of the semantic task, to investigate the effect of modality (pictures versus words) and difficulty (weak versus strong associates) but no patterns of lateralisation along the principal gradient were related to these subtler aspects of behaviour.

#### Supplementary analysis of the second gradient

While our main focus is on the principal gradient, we provide a supplementary analysis of the second gradient as described in Margulies et al. (2016), which captures the difference in connectivity between visual and motor networks. We show the means per hemisphere for Gradient 2, along with group means for each of the 400 parcels from Schaefer et al. (2018), and the 17 networks from Yeo et al. (2011). In this analysis, we characterise hemispheric differences per network for this gradient. Lastly, we provide bootstrapping analyses of the left versus right hemisphere network differences and establish which networks survive correction for multiple comparisons. All of these analyses follow the methods described above, with the results presented in Supplementary Analysis: Gradient 2, Supplementary Figs. 2–5.

### Study 2

#### Parametric manipulations of semantic control and working memory load

Previous research has shown the controlled retrieval of semantic information elicits activation within a highly left-lateralised semantic control network (Gonzalez Alam et al. 2019; Jackson 2021; Noonan et al. 2013), which is at least partially distinct from the bilateral multiple demand network that supports other aspects of cognitive control (Davey et al. 2016; Gao et al. 2021; Gonzalez Alam et al. 2018; Krieger-Redwood et al. 2015). Having established in Study 1 that individual differences in semantic cognition were associated with the magnitude of hemispheric differences on the principal gradient within a particular control network (Control-B), we then examined how semantic and non-semantic task demands modulate activation within this control network in Study 2.

We re-analysed an fMRI dataset (Gao et al. 2021) examining the effects of semantic control demands (via a parametric manipulation of strength of association) and verbal working memory load (using a parametric manipulation of the number of items to be maintained). In this new analysis, we extracted the effect of these parametric regressors on the BOLD response in left- and right-hemisphere components of large-scale networks that showed behavioural associations with lateralisation effects on the principal gradient in Study 1. Previous research has shown that retrieving semantic links between more weakly-related words elicits strong engagement of the left-lateralised semantic control network (Jackson 2021; Noonan et al. 2013). In contrast, higher loads in WM tasks are associated with greater responses within the Multiple Demand Network, particularly within the left hemisphere for verbal materials (Emch et al. 2019; Fedorenko et al. 2013). Our analysis, therefore, allowed us to compare the effects of task demands in domains associated with distinct control networks, with potentially different patterns of lateralisation. This experiment did not include a visual reasoning task comparable to Ravens Advanced Progressive Matrices, and consequently our focus was on comparing left-lateralised semantic cognition with another verbal yet non-semantic task. The tasks were broadly matched in terms of input processing and motor responses; however, the way in which control demands were manipulated was not identical across these tasks (i.e., difficulty of retrieval versus amount of information to be maintained).

We predicted a lateralised response to semantic but not non-semantic control demands specifically for networks in which individual differences in semantic cognition were associated with hemispheric differences in principal gradient values—i.e., a stronger response to semantic control than to working memory demands in the left but not right hemisphere within the Control-B network.

#### Participants

As reported in Gao et al. (2021), a group of 32 young healthy participants aged 19–35 (mean age = 21.97 ± 3.47 years; 19 females) was recruited from the University of York. They were all right-handed, native English speakers, with normal or corrected-to-normal vision and no history of psychiatric or neurological illness. The study was approved by the Research Ethics Committee of the York Neuroimaging Centre. All volunteers provided informed written consent and received monetary compensation or course credit for their participation. The data from one task was excluded for four participants due to head motion, and one additional WM dataset was excluded due to errors in recording the responses. The final sample included 28 participants for the semantic task and 27 participants for the WM task, with 26 participants completing both tasks.

#### Materials and procedure

The materials and procedure for this experiment (as well as the results reported in Sect. Study 2.) are described fully in Gao et al. (2021). We summarise below the aspects that are relevant to the present study.

##### Semantic task

In the on-line semantic task in fMRI, participants had to judge whether pairs of words were semantically related or unrelated. The stimuli were 192 English concrete noun word-pairs (abstract words and words drawn from the same taxonomic category were excluded, so that only thematic links were evaluated). The stimuli had parametrically varying degrees of thematic relatedness depending on their frequency of co-occurrence. The degree of relatedness was quantified using distributed representations of word meanings obtained from the word2vec neural network, trained on the 100 billion-word Google News dataset (Mikolov et al. 2013), defining the strength of the semantic relationship between pairs of words using the cosine similarity method. The stimulus set was manipulated so there was a parametric continuum of relatedness, from ‘not related at all’ to ‘strongly related’. Since the degree of relatedness was continuous, there were no clear ‘correct’ or ‘incorrect’ answers; instead, the trials were sorted according to whether participants judged each trial to be related or unrelated based on their own experience. Difficulty was then estimated by binning the stimuli into 5 categories for related and unrelated trials separately according to their word2vec score for each individual participant (since pairs judged to be related varied across participants). For trials judged to be related, a lower word2vec score was associated with increased difficulty (since establishing a semantic link for less strongly related items is harder); conversely, for trials judged to be unrelated, a higher word2vec score increased difficulty (since rejecting a relationship between associated words is harder). Each trial began with a visually presented word (1.5 s), followed by a central fixation (1.5 s), then the second word (1.5 s), followed by a 3 s response period where participants indicated whether the word was related or not pressing one of two buttons with their right hand. Participants performed 4 runs of this task, each lasting 7.3 min. Both response time and choice were recorded.

Word2vec has been shown to predict human behaviour better than other approaches such as latent semantic analysis (Pereira et al. 2016). Previous research has shown that semantic distance, as measured by word2vec, is negatively correlated with the strength of activation in the semantic control network: weakly-related trials require more controlled retrieval to identify a semantic link (Hoffman 2018; Teige et al. 2019), engaging well-defined regions of the semantic control network, including left inferior frontal gyrus (Zhang et al. 2021); this allows us to use word2vec scores as a proxy for semantic control demands (Badre et al. 2005; Teige et al. 2019; Wagner et al. 2001).

##### Working memory task

Non-semantic control demands were manipulated in a verbal working memory task, using a parametric manipulation of the number of items participants had to maintain in memory. This task had a similar structure and method of presentation to the semantic task; each trial began with a letter string (3 to 7 letters) presented at the centre of the screen (for 1.5 s), followed by a fixation (for 1.5 s). Participants were asked to remember these letters. Next, two letters were shown on the screen (for 1.5 s) and participants judged whether both of them had been presented in the letter string by pressing one of two buttons (with this decision phase presented for 3 s). Participants were told that the order of the letters did not matter. The working memory load was manipulated by varying the number of letters memorised in each trial; there were five levels of load from 3 to 7 letters (to match the 5 levels of word2vec in the semantic task). Both response time and accuracy were recorded. Participants completed 3 runs, each containing 40 trials and lasting for 6.1 min.

#### Analysis

The univariate analysis of these parametric manipulations yielded effect maps for semantic control and working memory demands, reported in Gao et al. (2021), which we employed in a ROI analysis to examine which networks showed significant task differences across hemispheres. For the participant level analysis, we binarised the Yeo network maps that showed significant behavioural associations in Study 1 and used them as ROI masks to extract the percent signal change value for each of the 26 participants separately in the left and right hemispheres for each condition of the task (i.e., related and unrelated semantic judgements, and working memory) using the featquery tool in FSL 6. We entered these values for each participant into separate repeated-measures ANOVAs for each network, examining ‘Hemisphere’ and ‘Condition’ as within-subjects factors.

## Results

### Study 1

#### Gradient values for the Schaeffer parcellation in left and right hemispheres

**Fig. 2:**
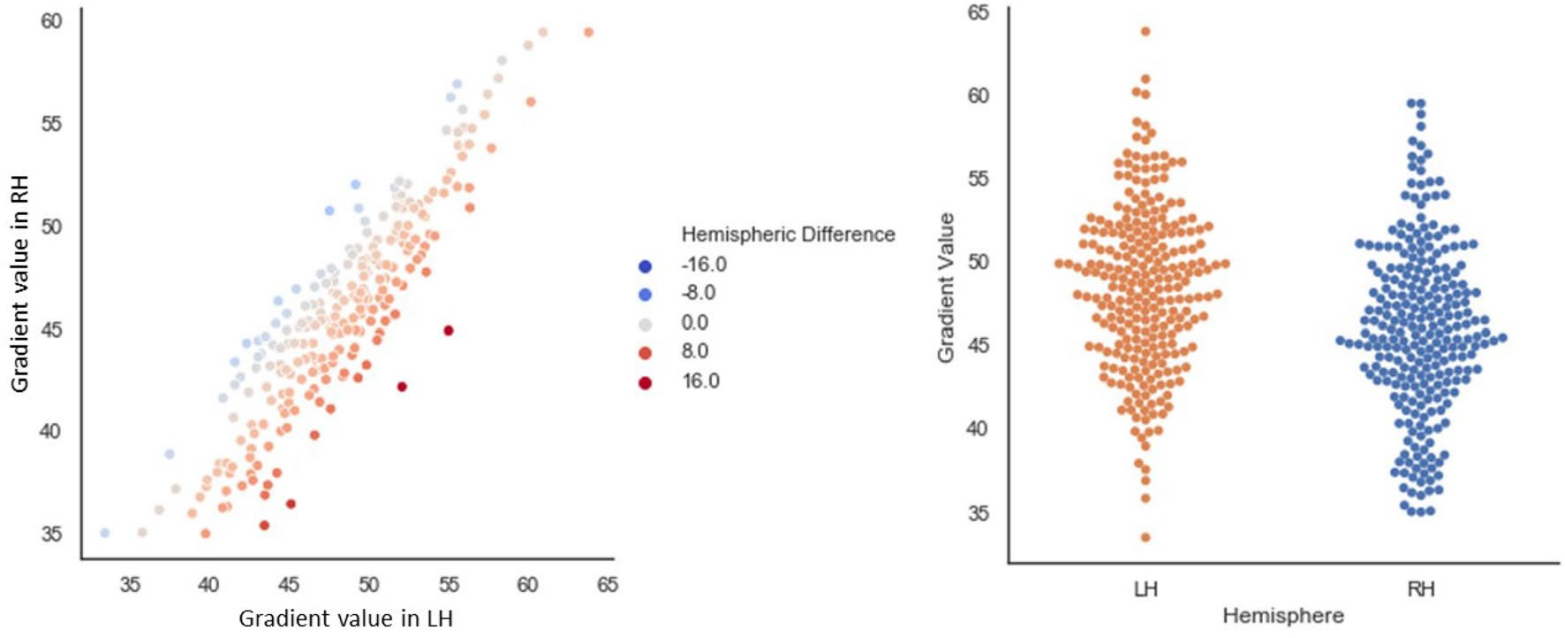
The left panel depicts a linear relationship in our sample’s mean left and right hemisphere values on the principal gradient. The ‘Hemispheric Difference’ legend of the scatterplot depicts the result of subtracting the LH–RH mean gradient loadings for the whole hemisphere per participant. Positive values reflect closer proximity to the heteromodal end of the gradient in LH. Negative values reflect closer proximity to the heteromodal end of the gradient in RH. The right panel depicts the distributions of mean global hemispheric values per participant in our sample. In both plots, each dot represents one participant. The scale on both plots indicates values on the principal gradient, which were re-scaled to range from 0 to 100

Our first analysis step revealed a global mean difference on the principal gradient (see Fig. 2), with higher values in the left hemisphere (paired samples *t* test: *t*(252) = 18.38, *p *< 0.001); the presence of higher global mean values in the left compared to the right hemisphere was observed in 87.7% of our sample (see the scatterplot in Fig. 2). Participants’ left and right hemisphere mean gradient values were very highly correlated (Pearson’s *r *= 0.9, *p *< 0.001), despite this global difference.

**Fig. 3:**
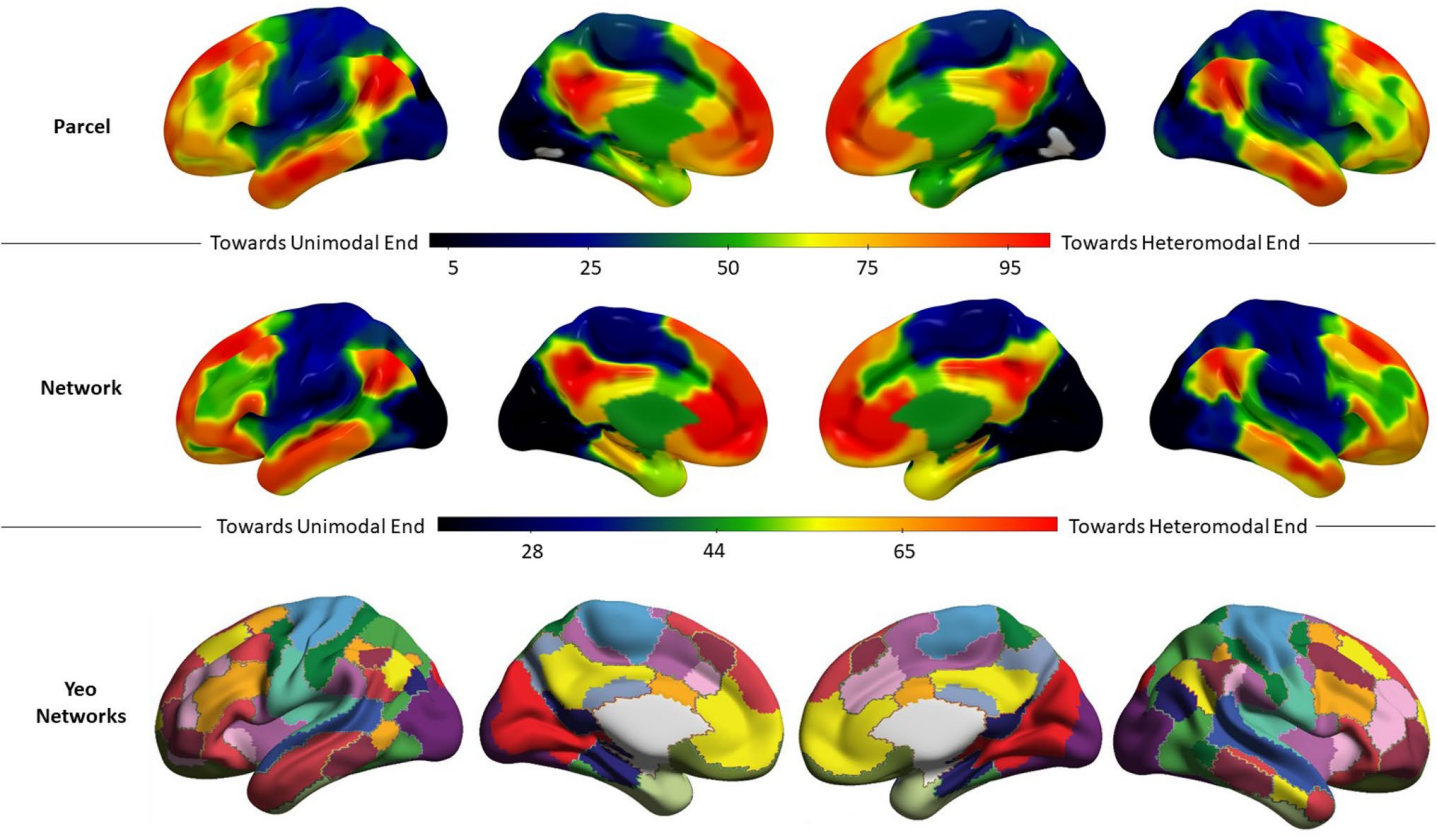
Top row: Group mean principal gradient value for each parcel in Schaeffer’s 400-parcel solution for our sample of 253 participants. Middle row: Group mean principal gradient value for each network in Yeo’s 17-network solution for our sample of 253 participants. Gradient units are arbitrary and have been normalised on a 0–100 minmax scale. Bottom row: 17 network parcellation by Yeo et al. (2011; the colour code followed in this figure replicates that of Buckner et al. 2011)

As expected, given our gradient alignment methods, the gradient decomposition of our 253-participant sample showed a principal gradient very similar to the one reported by Margulies et al. (2016), both at the parcel level (Fig. 3, top row) and at the network level (Fig. 3, middle row); see also Mckeown et al. (2020).

#### Hemispheric difference analysis at the network level

**Fig. 4:**
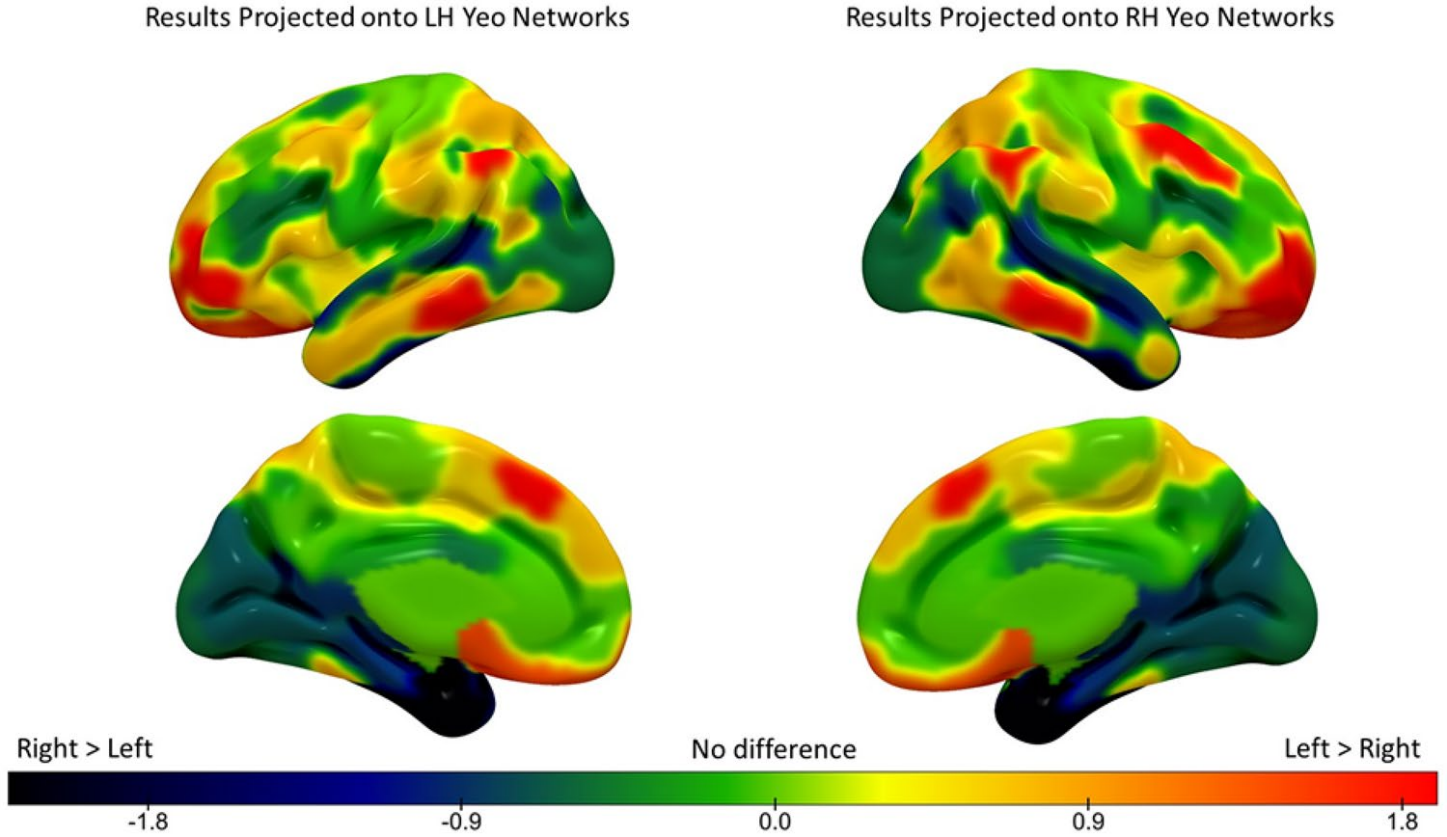
Hemispheric differences in principal gradient values across the 17 Yeo networks (z-scored). The warm colours represent principal gradient loadings that are nearer the heteromodal apex in LH compared to RH. Cool colours represent principal gradient loadings that are nearer the heteromodal apex in RH compared to LH

In order to compare the principal gradient loadings of the regions captured in the 400-region parcellation across the cerebral hemispheres, we averaged all parcels that fell within each network in the left and right hemispheres separately, and then performed a subtraction (left–right) and *z*-scored the resulting differences. The results can be seen in Fig. 4. The principal gradient loadings in warm colours are nearer the heteromodal apex in the left hemisphere compared to the right, and the cool colours represent principal gradient loadings that are nearer the heteromodal apex in the right hemisphere compared to the left. Since Yeo et al. (2011) networks are not symmetrical, subtracting left–right in one network will yield the same value for both hemispheres, but in different topological locations. To visualise this, in Fig. 4 we projected the results of the subtraction for each network (which are the same) onto the left and right hemisphere network maps (which are not the same). The value of these left–right network gradient differences was highly correlated with the principal gradient at the group level (*r *= 0.93, *p *< 0.0001), consistent with the expectation that heteromodal cortex shows more divergent connectivity across the hemispheres than unimodal cortex.

**Fig. 5:**
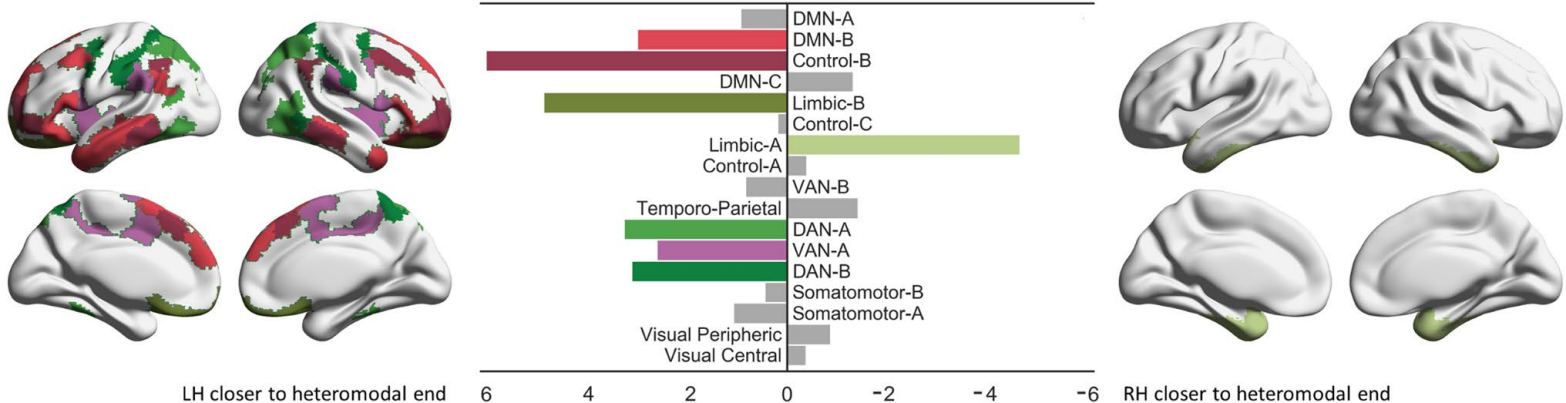
Results of permutation testing of LH versus RH positions on the principal gradient for each network (5000 simulations, Bonferroni-corrected alpha for 17 comparisons). The size of each bar reflects the normalized empirically observed mean difference across the hemispheres for each network. Coloured bars denote networks that showed significant differences (see Table 1 for exact *p* values) and are colour-coded to indicate the position of each network in the brain. The brain map on the left side of the plot shows networks that were closer to the heteromodal end of the gradient in LH, while the brain map on the right side of the plot shows one network that was closer to the heteromodal end of the gradient in RH

We performed a repeated-measures ANOVA to formally test for differences in principal gradient loadings at the network level (2 hemispheres by 17 networks), controlling for global hemispheric differences in gradient values by entering each participant’s global left–right difference value as a covariate of no interest. The results of this ANOVA revealed significant main effects of hemisphere [*F*(1, 251) = 538.82, *p *< 0.0001, *ηp*^2^ = 0.68], and network [*F*(8.48, 2127.52) = 902.44, *p *< 0.0001, *ηp*^2^ = 0.78], as well as a significant hemisphere by network interaction [*F*(10.62, 2664.95) = 18.61, *p *< 0.0001, *ηp*^2^ = 0.07; all values with Greenhouse–Geisser correction to account for violations of the sphericity assumption]. Subsequent post-hoc tests comparing the left and right hemispheres for each network (using permutation testing with 5000 simulations to establish significance; Bonferroni-corrected for 17 comparisons) revealed that these hemispheric differences were robust for seven networks: DMN-B, Control-B, Limbic-B, Limbic-A, DAN-A, DAN-B, and VAN-A (Fig. 5). Only these seven networks were carried forward for further analyses. Supplementary Fig. 1 shows the distribution of gradient values for these seven networks.

**Table 1:** Normalised (on a scale of 0–100) means across hemispheres for each Yeo network

Network	LH mean (SD)	RH mean (SD)	Corrected *p* value
DMN-A	82.76 (5.84)	81.85 (5.5)	> 0.1
DMN-B	77.62 (6.2)	74.69 (6.88)	.000***
Control-B	73.29 (7.51)	67.39 (7.44)	.000***
DMN-C	64.75 (9.98)	66.05 (10.4)	> 0.1
Limbic-B	67.51 (10.38)	62.74 (9.54)	.000***
Control-C	59.6 (12.14)	59.41 (11.38)	> 0.1
Limbic-A	54.66 (9.51)	59.22 (8.53)	.000***
Control-A	52.37 (7.54)	52.76 (7.59)	> 0.1
Salience/VAN-B	50.67 (9.18)	49.85 (9.18)	> 0.1
Temporo-parietal	48.74 (11.07)	50.14 (10.37)	> 0.1
DAN-A	39.03 (8.61)	35.84 (8.35)	.000***
Salience/VAN-A	35.94 (7.37)	33.38 (7.29)	.000***
DAN-B	34.11 (7.57)	31.07 (7.3)	.000***
Somatomotor-B	29.47 (7.33)	29.03 (7.38)	> 0.1
Somatomotor-A	29.56 (7.29)	28.51 (7.47)	> 0.1
Visual peripheric	18.84 (8.96)	19.69 (8.73)	> 0.1
Visual central	19.11 (6.57)	19.48 (7.04)	> 0.1

Larger values reflect greater proximity to the heteromodal end of the principal gradient. The *p* values indicate the results of pairwise bootstrapped permutation testing of LH vs RH principal gradient means for each network (5000 simulations)

*** = significant at *p *< 0.0002 with Bonferroni-correction for 17 comparisons

#### Behavioural regressions

**Fig. 6:**
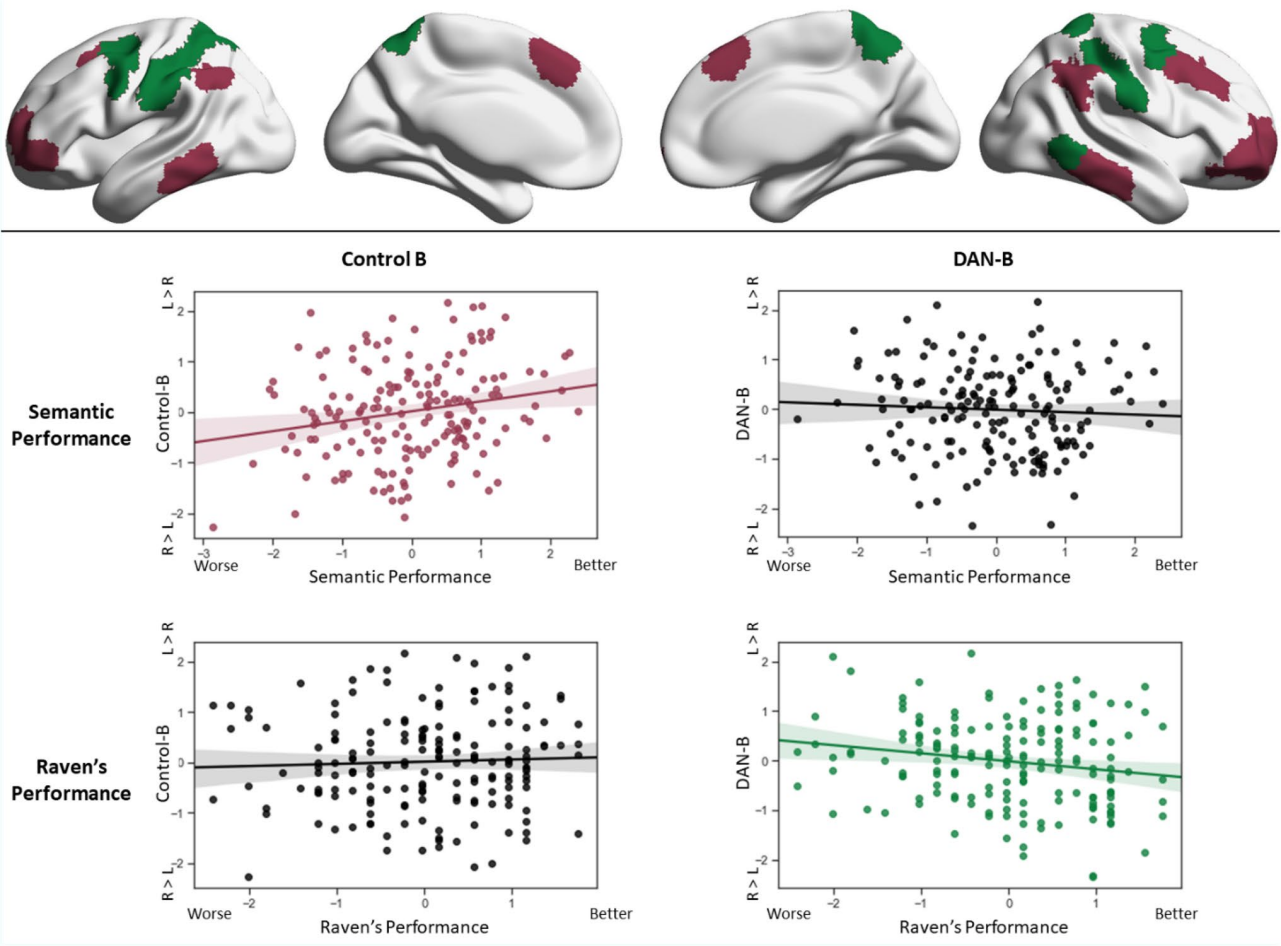
Scatterplots showing the relationship between hemispheric difference scores on the principal gradient and efficiency on semantic decisions (middle row) and accuracy on a visual reasoning task (Raven’s progressive matrices; bottom row). Only networks with significant results are shown (Control B on the left-hand side; DAN-B on the right-hand side). The scatterplots in colour denote significant effects in the regression model and have been colour coded to the networks driving the effect, shown in the top row

We next tested whether the degree of difference in principal gradient loadings across hemispheres for each Yeo network was associated with performance on semantic and non-semantic (visual reasoning and working memory) tasks outside the scanner. We defined regression models using the empirically observed mean hemispheric difference in gradient scores (left–right hemispheres) for each network as the dependent variable, and the efficiency of semantic decisions, maximum number of items remembered in digit span, and accuracy on Raven’s matrices as three explanatory variables per participant. The model also included efficiency in a perceptual matching task matched superficially to the semantic tasks. There was a significant association between task performance and hemispheric gradient differences for two out of seven networks (only networks showing a significant difference on the principal gradient in the analysis above were included). Hemispheric differences in gradient values for Control-B showed a positive association with overall semantic performance (*β* = 0.19, *p *= 0.02), and no relationship with working memory (*β* = 0.05, *p *> 0.1) or visual reasoning (*β *= 0.003, *p *> 0.1). DAN-B showed a negative association between left–right hemisphere gradient loadings and visual reasoning (*β* =  − 0.16, *p *= 0.02) and no relationship with semantic performance (*β* =  − 0.1, *p *> 0.1) or working memory (*β* =  − 0.13, *p *= 0.07) (see Fig. 6). In sum, participants whose Control-B network was closer to the heteromodal DMN end of the principal gradient in the left hemisphere compared with the right showed more efficient semantic retrieval; in contrast when the DAN-B network was closer to the heteromodal end of the principal gradient in the right hemisphere compared with the left, participants showed better visual reasoning on a matrices task. There were no significant associations with working memory. An additional analysis comparing subtasks of the semantic battery observed no significant association between gradient hemispheric differences and the effects of the modality of presentation (pictures versus words) or strength of association (weak versus strong associations), with all *p* values > 0.1 (see supplementary materials, Table S1); these results are in line with the PCA reported in Sect. “Materials”. (Semantic dimensionality reduction), showing that the variance of this task can be explained with one single factor. All these analyses controlled for differences between the semantic and non-semantic tasks by including a perceptual task matched in presentation and response requirements with the semantic task (see Sect. “Materials”: ‘Perceptual figure matching task’). The efficiency scores of this perceptual task showed a significant negative association with the DAN-B hemispheric difference scores (*β* = 0.19, *p *= 0.02), but no association with the Control-B hemispheric difference scores (*β* = 0.02, *p *> 0.1).

Given that individual differences in semantic cognition predicted hemispheric differences in principal gradient values, we next asked if Control-B and DMN networks are closer on the principal gradient of connectivity in the left hemisphere compared with the right. This finding would be consistent with the greater coupling of these networks in states of controlled semantic retrieval reported by Davey et al. (2016). Yet the sensitivity of the principal gradient to this pattern of functionally-relevant network similarity in the left hemisphere is not yet established since both Control-B and DMN-B (the adjacent network on the principal gradient) showed higher gradient values for the left hemisphere compared with the right in the analysis in Fig. 5.

We computed the distance on the principal gradient between Control-B and the two networks that were closer to the heteromodal apex of the principal gradient (DMN-A and DMN-B) for each hemisphere separately and compared these distances across hemispheres using paired t-tests (all p values reported are corrected for multiple comparisons). There was a significantly smaller difference in principal gradient values between Control-B and DMN-B in the left hemisphere compared with the right (mean difference in LH = 4.33, SD = 7.4; and in RH = 7.29, SD = 7.93; *t*(174) = 6.52, *p *< 0.001). There was a similarly smaller difference in principal gradient values between Control-B and DMN-A in the left hemisphere compared with the right (mean difference in LH = 9.47, SD = 7.77; and in RH = 14.45, SD = 7.06; *t*(174) = 13.07, *p *< 0.001). This confirms that Control-B is closer to DMN along the principal gradient.

**Fig. 7:**
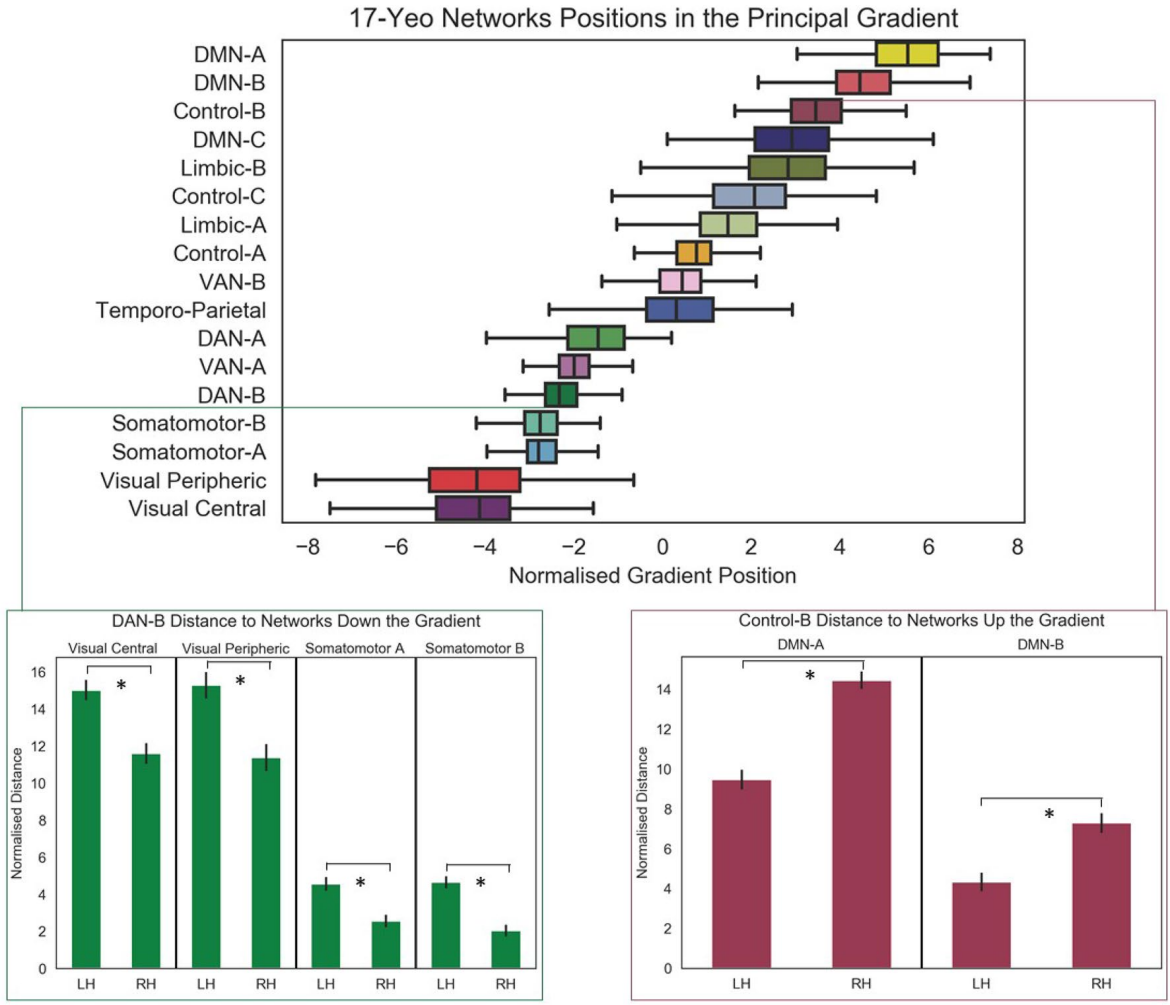
Differences of network positions in the principal gradient between pairs of networks that are relevant for lateralised cognitive processes. * = *p *< .001 (corrected for multiple comparisons)

We repeated this analysis to establish if DAN-B has greater proximity to sensorimotor networks in the right hemisphere compared with the left. There were significantly smaller gradient distances in the right hemisphere compared with the left for all four relevant network comparisons: (i) visual central: LH = 15.01, SD = 8.7; RH = 11.6, SD = 9.09; *t*(174) = 7.96, *p *< 0.001); (ii) visual peripheral: LH = 15.28, SD = 11.33; RH = 11.37, SD = 11.47; *t*(174) = 8.76, *p *< 0.001), (iii) somatomotor-A (LH = 4.55, SD = 5.93; RH = 2.55, SD = 5.26; *t*(174) = 7.95, *p *< 0.001) and (iv) somatomotor-B (LH = 4.64, SD = 5.12; RH = 2.04, SD = 4.88; *t*(174) = 9.75, *p *< 0.001). DAN-B was closer to all sensorimotor networks in the right hemisphere compared with the left. These results can be seen in Fig. 7.

### Study 2

#### Behavioural results

As reported in Gao et al. (2021), equal numbers of word pairs were judged to be related or unrelated by the participants in the semantic task [mean ratio 0.491 vs. 0.495, *χ*^2^(1) = 0.00021, *p *> 0.995]. Linear mixed-effects models examined whether associative strength and WM load were reliable predictors of behaviour. Gao et al. showed that both the strength of the semantic association (word2vec value) and WM load successfully manipulated task difficulty. For the semantic task, the continuous word2vec value was positively associated with a higher probability that participants would identify a semantic relationship between the words [*χ*^2^(1) = 2421.3, *p *< 0.001] in a logistic regression. When word pairs were grouped into 5 levels according to their word2vec value, the relationship was still significant [*χ*^2^(1) = 2467.8, *p *< 0.001].

Regarding the validity of the novel manipulation of semantic control used in this task, linear mixed effects models showed that association strength modulated RT in a manner consistent with the deployment of semantic control: trials in the ‘related’ category with higher word2vec scores should involve less effort, since there is a readily available context, whereas those with lower word2vec scores require control for establishing a context; the reverse pattern is expected for trials judged to be ‘unrelated’, where a high word2vec score means the context must be effortfully suppressed, while a low word2vec score makes the link easier to reject, since no shared context exists. Linear mixed-effects analyses, including participant as a between-subject variable and association level as a within-subject variable, showed associative strength was negatively associated with RT for related trials [*χ*^2^(1) = 146.6, *p *< 0.001], and positively associated with RT for unrelated trials [*χ*^2^(1) = 58.668, *p *< 0.001]. It was more difficult for participants to retrieve a semantic connection between two words when the strength of association was lower; on the contrary, it was easier for them to decide there was no semantic connection between word pairs with low word2vec values.

For the WM task, the proportion of correct responses was 84.8%, when all memory load levels were considered. The more items to be maintained or manipulated in WM, the more difficult the trial was expected to become. A logistic regression showed that higher WM load was associated with lower accuracy [*χ*^2^(1) = 112.4, *p *< 0.001]. A further linear mixed-effects model with participant as a between-subject variable and memory load as a within-subject variable revealed a significant positive relationship between load level and RT for correct responses [*χ*^2^(1) = 39.826, *p *< 0.001].

A two-way repeated-measures ANOVA examining the effects of task condition (‘related’, ‘unrelated’ and ‘WM correct’) and difficulty level (5 levels) on the proportional change in RT for each difficulty level of the task, relative to the average RT for each condition, showed a significant interaction between task condition and difficulty level [*F*(5.40, 134.88) = 8.33, *p *< 0.001, Greenhouse–Geisser corrected] along with a main effect of difficulty level [*F*(3.13, 78.35) = 53.26, *p *< 0.001, Greenhouse–Geisser corrected]. Together, these results suggest that association strength and WM load successfully manipulated task difficulty, engaging effortful cognition. Summary measures of the behavioural data reported here are provided in Supplementary Table 6 and Supplementary Fig. 10.

#### Parametric effects of control demands

Study 1 found that when Control-B is closer to the DMN-end of the principal gradient in the left hemisphere versus the right, participants have more efficient semantic retrieval. In contrast, when DAN-B is closer to the heteromodal end of the principal gradient in the right hemisphere, participants show better visual reasoning on a progressive matrices task. These findings predict a hemispheric dissociation between networks in the effects of control demands across domains (i.e., in effects of semantic control demands and non-semantic difficulty—even within the verbal domain). In Study 2, we tested this prediction by examining the effects of parametric manipulations of semantic control demands (strength of association) and verbal working memory load on activation within the left and right hemisphere components of control-B and DAN-B networks (this data set did not include a visual reasoning task). An omnibus ANOVA examining the factors of hemisphere (left vs. right), task difficulty (related semantic, unrelated semantic and working memory) and network (Control-B vs. DAN-B) showed a three-way interaction between these factors [*F*(2,54) = 6.71, *p *= 0.003].

**Fig. 8:**
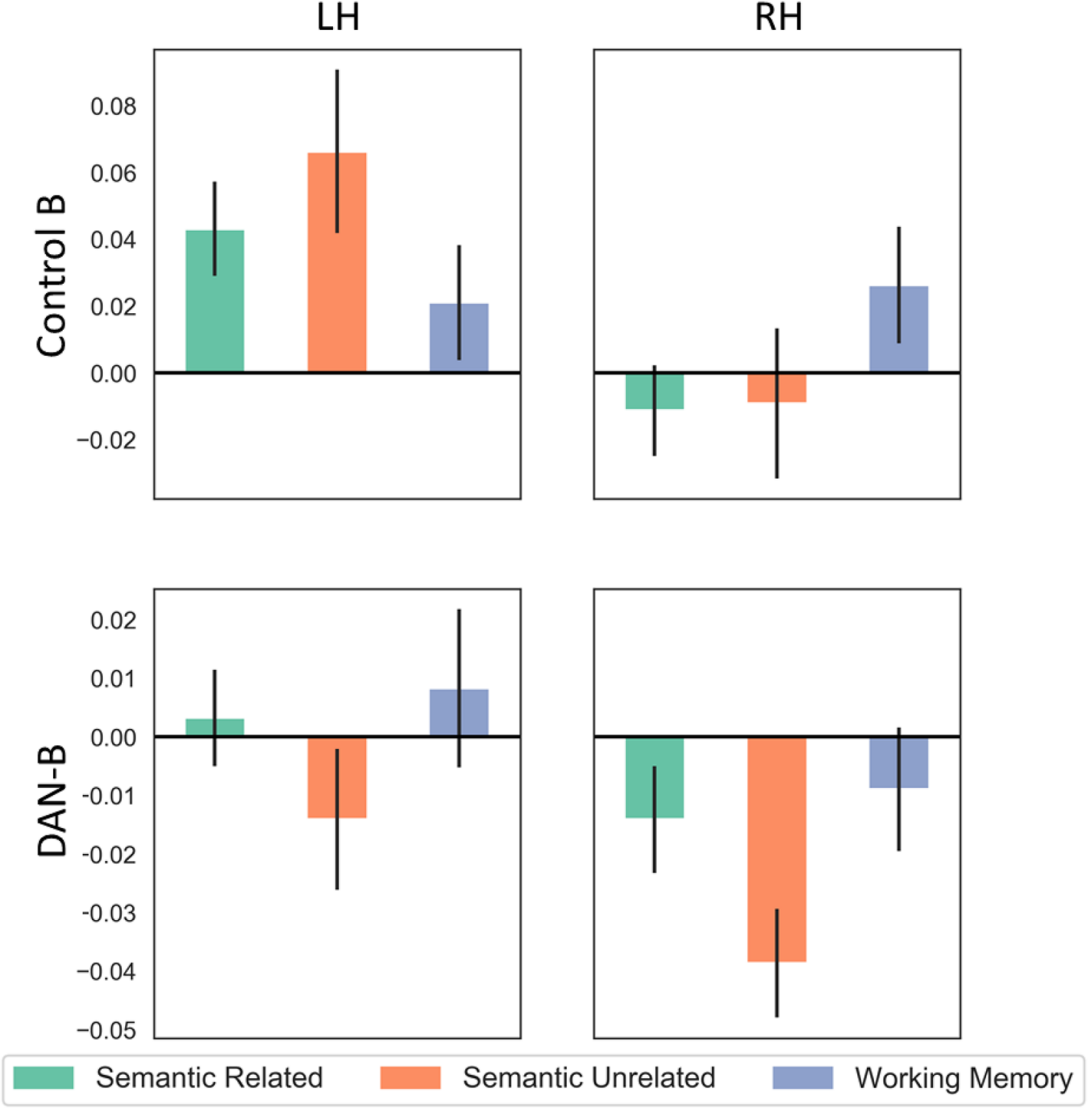
Parametric effects of difficulty in semantic and non-semantic tasks for 26 participants in Control B and DAN-B Yeo networks, split by hemisphere (the error bars depict the standard error of the mean), with effects expressed in percentage signal change

Separate repeated-measures ANOVAs for control-B and DAN-B found distinct patterns. Control-B showed a significant interaction between hemisphere and condition, reflecting larger effects of control demands in the semantic task relative to working memory [*F*(1.34, 36.07) = 7.72, *p *= 0.005, *ηp*^2^ = 0.22]. Post-hoc tests revealed a greater response to difficulty for semantic decisions in the left hemisphere versus the right (*p *< 0.001); in contrast, there were no hemispheric differences in the effect of WM load. There was no task by hemisphere interaction for DAN-B (*F* < 1). There were also no main effects of task in either network, but there was more activation in the left hemisphere overall, likely reflecting the verbal nature of the tasks. Full ANOVA results are reported in Table 2. These results confirm that the left-lateralised components of Control-B show a specific response to semantic control demands, but not to working memory load. In contrast, DAN-B shows no difference in response to these two forms of verbal control across hemispheres. These effects are shown in Fig. 8.

**Table 2:** Results of the ANOVAs on the parametric difficulty effect maps

Model	Effect	*df*	*F*	*p*	*ηp*^2^
Omnibus	Interaction^+^	1.55,41.85	6.71	0.006	0.199
	Task*hemisphere^+^	1.35,36.4	5.07	0.021	0.158
	Task*network	2,54	1.42	0.25	0.05
	Hemisphere*network	1,27	6.81	0.015	0.201
	Hemisphere	1,27	37.9	< 0.0001	0.584
	Network	1,27	9.59	0.005	0.262
	Task	2,54	0.26	0.77	0.89
Control-B	Interaction^+^	1.33,36.07	7.72	0.005	0.222
	Hemisphere	1,27	27.23	< 0.0001	0.502
	Task	2,54	0.16	0.851	0.006
DAN-B	Interaction^+^	1.62,43.62	0.21	0.768	0.008
	Hemisphere	1,27	18.49	< 0.0001	0.406
	Task	2,54	2.25	0.116	0.077

The effects marked with + were subjected to Greenhouse–Geisser corrected since our data violated the assumption of sphericity (Mauchley’s test of sphericity *p *< .05 in both cases)

## Discussion

This study investigates the lateralisation of function along the principal gradient—a key topographical component of large-scale intrinsic connectivity that captures the separation of unimodal and heteromodal cortex (Margulies et al. 2016). We show that intrinsic connectivity patterns in the two hemispheres are situated at different points along the principal gradient: overall, left hemisphere parcels are closer to the heteromodal end of the principal gradient than right hemisphere parcels, consistent with the role of this hemisphere in key heteromodal functions, such as semantic cognition and language. This pattern was observed in many canonical heteromodal networks derived from a whole-brain parcellation of resting-state data (Yeo et al. 2011), including control, default, dorsal and ventral attention networks; however, this pattern was inverted for Limbic-A, centred on the ventral anterior temporal lobe (ATL). There was also no gradient difference between the hemispheres in sensorimotor networks. In Study 1, individual differences in the relative gradient positions of networks across the hemispheres were found to have functional associations with two cognitive processes with opposing patterns of lateralisation, semantic cognition and visual reasoning (there were no effects for working memory). Participants whose Control-B network was closer to the heteromodal DMN end of the principal gradient in the left hemisphere compared with the right showed more efficient semantic retrieval; in contrast when the DAN-B network was closer to the heteromodal end of the principal gradient in the right hemisphere compared with the left, participants showed better visual reasoning on a progressive matrices task. Finally, in Study 2, we established that Control-B dissociates from DAN-B in the effect of verbal task demands on task activation in the left and right hemispheres. Control-B shows a left-lateralised response to semantic control demands but not working memory load, consistent with the view that lateralised control regions near the DMN apex of the principal gradient support controlled semantic retrieval.

To date, only one previous study has attempted to describe hemispheric differences in the principal gradient (Liang et al. 2021). Despite important differences in methodology, our findings align with Liang et al.’s study: both investigations found higher gradient values in the left than right hemisphere for ventromedial prefrontal cortex, IFG and lateral ATL. However, Liang et al. extracted separate gradients for the left and right hemispheres and considered these patterns within a 7-network parcellation; consequently, they could not identify the sub-network hemispheric differences that we observed, or directly compare left and right hemisphere networks within the same decomposition. The study by Liang et al. also did not assess the functional significance of hemispheric differences on the principal gradient, which was the main focus of the current study.

We found that left hemisphere parcels were, in general, closer than right hemisphere parcels to the DMN apex of the principal gradient, helping to explain why key heteromodal functions—such as language and semantic cognition—are left-lateralised. Margulies et al. (2016) found that the terms language:syntax and language:semantics were among the BrainMap behaviour terms closest to the heteromodal end of the principal gradient; similarly, verbal semantics was towards the heteromodal apex in Neurosynth (a meta-analytic tool; Yarkoni et al. 2011). Language and semantics both depend on the retrieval of heteromodal representations—extracted from diverse sensory-motor features when we acquire concepts and words; moreover, they both require retrieval to be controlled to fit rapidly changing goals and contexts. These different components of semantic cognition—conceptual representations plus control processes—are lateralised to different degrees (Gonzalez Alam et al. 2019). Semantic control processes are supported by a strongly left-lateralised network, encompassing left inferior frontal gyrus, and left posterior middle and inferior temporal cortex (Jackson 2021; Noonan et al. 2013). The resting-state functional connectivity between these semantic control sites is stronger in the left hemisphere compared with the right (Gonzalez Alam et al. 2019). In contrast, heteromodal conceptual representation is thought to be supported by bilateral ventral ATL (Ding et al. 2020; Lambon Ralph et al. 2017; Patterson et al. 2007). Evidence for bilateral conceptual representation in ventral ATL is provided by neuroimaging studies (Bright et al. 2004; Tranel et al. 2005; Vandenberghe et al. 1996; Visser et al. 2009, 2011) and neuropsychology; patients with bilateral ventrolateral ATL damage show severe semantic impairment (for example, in semantic dementia), while patients with unilateral lesions have milder deficits (Rice et al. 2018).

This difference between strongly lateralised semantic control processes and bilateral conceptual representations may help to explain why Limbic-A, centred on the ventral anterior temporal lobe, was situated closer to the DMN end of the principal gradient in the right hemisphere compared with the left. Gonzalez Alam et al. (2019) found that right ATL was more connected to core DMN regions, including angular gyrus and dorsomedial prefrontal cortex; in contrast, left ATL was more connected to left-lateralised sites implicated in semantic control, including left intraparietal sulcus and left anterior insula bordering ventral parts of inferior frontal gyrus. In the left hemisphere, the principal gradient captures the order of networks from DMN, through the semantic control network, to executive regions (Wang et al. 2020). As a consequence, this proximity (and shared connectivity) of left ATL to semantic control regions might explain the unique gradient difference in Limbic-A. Right hemisphere components of this network might be closer to the heteromodal apex of the principle gradient because they are further from left-lateralised control networks situated towards the middle of the gradient.

The left-lateralised semantic control network is thought to be partially distinct from multiple demand cortex that responds to executive demands across domains: for example, effects of semantic but not non-semantic control demands are observed in anterior aspects of inferior frontal gyrus and posterior middle temporal gyrus (Davey et al. 2015, 2016; Hoffman et al. 2010; Jackson 2021; Noonan et al. 2013; Whitney et al. 2011, 2012). Similarly, the frontoparietal control network, defined through analyses of intrinsic functional connectivity, shows a bipartite organisation (Dixon et al. 2018), overlapping with Control-A and Control-B networks within the Yeo et al. (2011) parcellation used in this study. Dixon et al.’s (2018) control subnetwork including more anterior parts of both inferior prefrontal cortex and middle temporal gyrus has a topographical distribution that is similar to the functionally defined semantic control network (Jackson 2021; Noonan et al. 2013), and shows stronger interactions with DMN regions than the other control subnetwork. Similarly, the functionally defined semantic control network shows relatively strong intrinsic connectivity to both DMN, associated with heteromodal integration or abstraction, and domain-general executive and attention networks (Davey et al. 2016). This pattern of connectivity may allow states of controlled semantic cognition in which ongoing activation within DMN regions is shaped through the application of goal representations within the executive cortex to promote more weakly encoded aspects of knowledge (Wang et al. 2020). This finding is consistent with our observation of more efficient semantic cognition when the Control-B network was closer on the principal gradient to DMN in the left hemisphere as opposed to the right. Gradient differences between the two hemispheres might allow one control subnetwork to connect more strongly with DMN, supporting semantic control in the left hemisphere, while the other control subnetwork in the right hemisphere connects more strongly with sensory-motor regions, with advantages for demanding tasks that are oriented towards external sensory-motor features. This possibility is consistent with Wang et al. (2014) who found that control network regions in the left hemisphere have stronger connectivity with DMN, while right hemisphere control sites are closer in connectivity to attentional networks.

Like the frontoparietal regions linked to cognitive control, DMN also has subnetworks; this study provides some evidence that these subdivisions within control and DMN networks are functionally related. Just as we found a control network that was closer to the heteromodal end of the principal gradient in the left hemisphere, DMN-B (the adjacent network), showed the same pattern. DMN-B includes regions such as lateral ATL, angular gyrus, inferior frontal gyrus and dorsomedial prefrontal cortex that are associated with semantic processing in the left hemisphere (Jackson 2021; Jefferies 2013; Lambon Ralph et al. 2017; Noonan et al. 2013; Rice et al. 2015b), and this DMN variant has repeatedly shown functional dissociations with core DMN regions such as posterior cingulate cortex and more ventromedial prefrontal regions (Chiou et al. 2020; Zhang et al. 2020), referred to here as DMN-A. DMN-B is associated with lateralised cognitive processes, like language and semantics, as well as social cognition (Andrews-Hanna et al. 2014). This network shows responses to externally generated, conceptual tasks, including those that interface with perception. In contrast, DMN-A or core DMN is thought to be more detached from perception, and is engaged by internally generated, self-referential and autobiographical memory processing (Chiou et al. 2020). It is interesting to note that it is DMN-B, not core DMN, that shows a lateralised position on the principal gradient. This is consistent with the possibility that lateralisation reflects the need to sustain and/or control heteromodal semantic retrieval (as opposed to the need to support internally-generated mental states, which are also associated with the heteromodal end of the principal gradient).

We found evidence of significant differences in lateralisation patterns within attentional networks as well, with both DAN and VAN falling closer to the heteromodal end of the gradient in the left hemisphere. Although attention has been traditionally conceptualised as a right-lateralised cognitive function, contemporary neuroscientific research paints a more nuanced picture with complex patterns of lateralisation across the traditionally accepted ventral and dorsal attention networks (Corbetta and Shulman 2002; Jeong and Xu 2016; Szczepanski et al. 2010; Thiebaut de Schotten et al. 2011a, b). Critically, the DAN also plays a role in the flexible coupling of the control network across hemispheres and subdivisions (Dixon et al. 2018; Wang et al. 2014). Both DAN and control networks showed significant but opposing behavioural associations in our individual differences’ analysis of the position of networks on the principal gradient across hemispheres. Hemispheric differences in DAN-B were related to Raven’s matrices performance, but in contrast to semantic cognition, participants whose DAN-B was closer to the heteromodal end of the gradient in the right hemisphere were better at the task. Performance in reasoning tasks relies on efficient interregional communication within the bilateral multiple-demand system, and between this control network and other regions, for example, areas that maintain visuo-spatial representations, to orchestrate complex cognition (Gläscher et al. 2010; Shin and Jeon 2021). Moreover, previous research has linked performance on progressive matrices to attentional capacity (Schweizer and Moosbrugger 2004), and performance can also be decomposed into in two components relating to perceptual and executive attention (Ren et al. 2012), with the latter corresponding more closely to the DAN (Corbetta et al. 2008; Corbetta and Shulman 2002) and accounting for more variance in visual reasoning tasks (Ren et al. 2013). The right hemisphere is particularly activated during the performance of this task in certain conditions (Bishop et al. 2008; Prabhakaran et al. 1997). Contrasting specific types of reasoning tasks, like matrix and analogical reasoning, reveals greater right-lateralised responses in fronto-parietal regions for matrix reasoning (Hobeika et al. 2016). Right frontal regions in this network also show a greater response as matrix tasks increase in complexity (Krawczyk et al. 2010a, b, 2011). Consequently, higher DAN gradient values in the right hemisphere might reflect closer integration of DAN and control networks in the right hemisphere, which facilitates the efficient deployment of attention to solve spatial relational reasoning problems.

We also compared semantic cognition with verbal WM tasks and found no lateralisation effects within these networks for the latter. This suggests that the lateralisation of semantic cognition is related to the controlled retrieval of conceptual representations as opposed to the need to use language in the task, and is compatible with accounts of WM demands engaging bilateral multiple demand cortex (Duncan, 2001, 2010; Fedorenko et al. 2013; Hugdahl et al. 2015). However, we do not provide a full description of lateralisation in verbal working memory, since Study 1 did not find any associations between digit span and hemispheric gradient differences for any networks; consequently networks relevant to working memory were not selected for analysis in Study 2.

There are several limitations of the current study. The tasks compared in Study 1 (semantic battery versus digit span and Raven’s progressive matrices) varied in multiple ways, including mode of response, task instructions, time allowed to respond and level of demand. In the analysis examining the association between individual differences in gradient lateralisation and semantic performance, we statistically controlled for performance on a visual matching task with similar input and response characteristics to the semantic task, helping to ensure that semantic task effects reflected the requirement for conceptual retrieval. The fact that we observed an association with hemispheric differences in gradient position in DAN-B but not in Control-B with this perceptual task, while the association of hemispheric differences in Control-B with semantic performance remained significant, makes it unlikely that differences between the semantic and non-semantic tasks gave rise to our results. However, there were no control tasks for digit span or visual reasoning; moreover, hemispheric differences in the principal gradient might be related to other non-semantic cognitive domains not assessed here. Furthermore, the parametric manipulations of difficulty in WM and semantic judgements in Study 2 are not analogous since we manipulated working memory load (i.e., items to be maintained) and the strength of the semantic association (i.e., semantic distance as measured by word2vec). As noted by Gao et al. (2021), the WM task was associated with faster responses than the semantic control task, perhaps because word reading takes longer than letter identification, but reading times are not necessarily relevant to the activation of control networks. Another difference among these tasks was that strength of association had a larger effect on RT than working memory load, although RT does not provide a direct measure of cognitive control demands. We selected these manipulations because the literature shows that they robustly vary the activation of control regions (Noonan et al. 2013; Jackson 2021; Fedorenko et al. 2013; Emch et al. 2019); however, it would also be possible to manipulate control demands in a more comparable way across these domains, for example, by varying the strength of the distractors in both tasks.

Our methods also did not allow us to investigate the source of the network asymmetries at the sub-network or parcel level, since the parcellation we used (Schaefer et al. 2018) did not provide homotopic regions that could be compared (see Popovych et al. 2021, for the effect of parcellation choice on resting-state results). The parcels we used from Schaefer et al. (2018) are derived separately for each hemisphere, and there are an unequal number assigned to each network across hemispheres. For example, the DAN-B network has 13 parcels in the left hemisphere organised in three subdivisions (postcentral, frontal eye fields and precentral ventral region), while it has only 11 in the right hemisphere (lacking the precentral ventral region and sporting only 8 postcentral parcels, opposed to 9 in the left hemisphere). These differences might give rise to local resting-state functional connectivity gradients that are present in one hemisphere but not the other (Gordon et al. 2016), and these differences in local organisation could have functional significance. Future research could therefore seek to verify these patterns using symmetrised parcellations (Glasser et al. 2016; Joliot et al. 2015), or use methods that exploit voxel-level timeseries homotopy (Gotts et al. 2013; Jo et al. 2012).

Another limitation is shared by many studies that employ dimensionality reduction methods: the number of dimensions retained for analysis is somewhat arbitrary (Supplementary Figure S6 shows that there is no clear plateauing of the eigenvalues). Here we focussed on the asymmetry of the principal gradient, as it captures the most variance and is known to be important for cognition as well as the organisation of large-scale networks on the cortical surface (Murphy et al. 2018, 2019; Turnbull et al. 2020; Wang et al. 2020). We also provide a supplemental analysis of gradient asymmetries in Gradient 2, but we opted not to extend the analysis to gradients explaining less variance as their interpretability is expected to be lower; moreover, studies have shown functional associations with Gradients 1 and 2, but not with Gradient 3 and beyond (Hong et al. 2019; Murphy et al. 2018, 2019; Turnbull et al. 2020; Wang et al. 2020). Future research could take a different approach by extracting a very large number of gradients, and then identify lower-order gradients that specifically capture hemispheric differences in higher-order gradients (see Valk et al. 2020).

Finally, it remains unclear why attentional networks (DAN-A; DAN-B and VAN-A) were closer to the heteromodal end of the principal gradient in the left hemisphere, even when the opposite pattern for DAN-B (closer proximity to heteromodal cortex in the right hemisphere) was associated with better visual attention. One possibility is that these attention networks can also support controlled semantic cognition, to varying degrees across people, and that these patterns of left-lateralised and right-lateralised connectivity are in competition. Future research could test whether the position of networks along the principal gradient relates to their capacity for efficient interaction, and whether there are differences in physical distance along the cortical surface in the two hemispheres that reflect the connectivity gradient differences we described.

## Conclusions

We found that networks associated with higher-order cognition in the left hemisphere are positioned closer to the heteromodal end of the principal gradient, including the DMN, control, limbic and attentional networks; in contrast, there were no differences in sensorimotor networks, in line with the literature on functional homotopy. The control-DAN dissociation we observed is compatible with recent proposals of a “inward-outward” organisational principle for control networks that differs across the hemispheres, with a privileged interaction of DMN-B and Control-B in the left hemisphere (Dixon et al. 2018; Wang et al. 2014). Individual difference analysis showed that relative network position across the hemispheres is associated with the efficiency of lateralised cognitive processes: proximity of DMN to control regions in the left hemisphere was associated with better semantic processing, while the proximity of DAN to control regions in the right hemisphere was associated with better visual reasoning. Analysis of task-based fMRI data in a separate study showed differential recruitment of the Control-B network across the hemispheres in response to semantic demands but not working memory load.

## Supplementary Information

Below is the link to the electronic supplementary material.

Supplementary file1 (DOCX 2111 KB)

## Data Availability

Gradient maps one to ten from the group-averaged dimension reduction analysis described in Sect. Neuroimaging below are publicly available on NeuroVault in a collection (https://neurovault.org/collections/6746/). Raw fMRI and questionnaire data are restricted in accordance with ERC and EU regulations.

## Code availability

All code used in the production of this manuscript is available from the corresponding authors upon reasonable request.
