# Supplementary material for: A tale of two gradients: differences between the left and right hemispheres predict semantic cognition
Source: Brain Struct Funct. 2021 Sep 12;227(2):631–54. doi: 10.1007/s00429-021-02374-w (PMC8844158; doi:10.1007/s00429-021-02374-w)
Supplement: Supplementary file 1 — Supplementary file1 (DOCX 2111 KB) [file 429_2021_2374_MOESM1_ESM.docx]

**Supplementary Materials**

**Study 1**

**Individual Difference Analysis of modality and difficulty in the semantic task**

We performed a supplementary analysis as part of the individual differences approach in Study 1. After finding an association between hemispheric differences in gradient position for the Control-B network and semantic performance, we asked whether these differences were related to the degree of semantic control demanded by the stimuli and/or to the modality of presentation. We defined a regression model where we entered as EVs the subtraction of each participant’s efficiency score in the ‘Weak’ – ‘Strong’ conditions (difficulty) and the subtraction of their ‘Word’ – ‘Picture’ scores (modality). This analysis yielded no significant results. The results can be consulted in Supplementary Table S1.

| **Network** | **Contrast** | **β** | **P (uncorrected)** |
| --- | --- | --- | --- |
| Control-B | Word > Picture | .09 | .23 |
|  | Strong > Weak | -.06 | .55 |
| DMN-B | Word > Picture | .08 | .26 |
|  | Strong > Weak | .01 | .87 |
| DAN-A | Word > Picture | .04 | .62 |
|  | Strong > Weak | .11 | .21 |
| DAN-B | Word > Picture | .05 | .52 |
|  | Strong > Weak | .11 | .2 |
| Limbic-B | Word > Picture | -.01 | .88 |
|  | Strong > Weak | -.08 | .42 |
| Limbic-A | Word > Picture | .05 | .55 |
|  | Strong > Weak | .15 | .11 |
| VAN-A | Word > Picture | .01 | .89 |
|  | Strong > Weak | .01 | .89 |

Table S1. OLS Regression results for semantic task contrasts.

**Distribution of Gradient Values per Network**


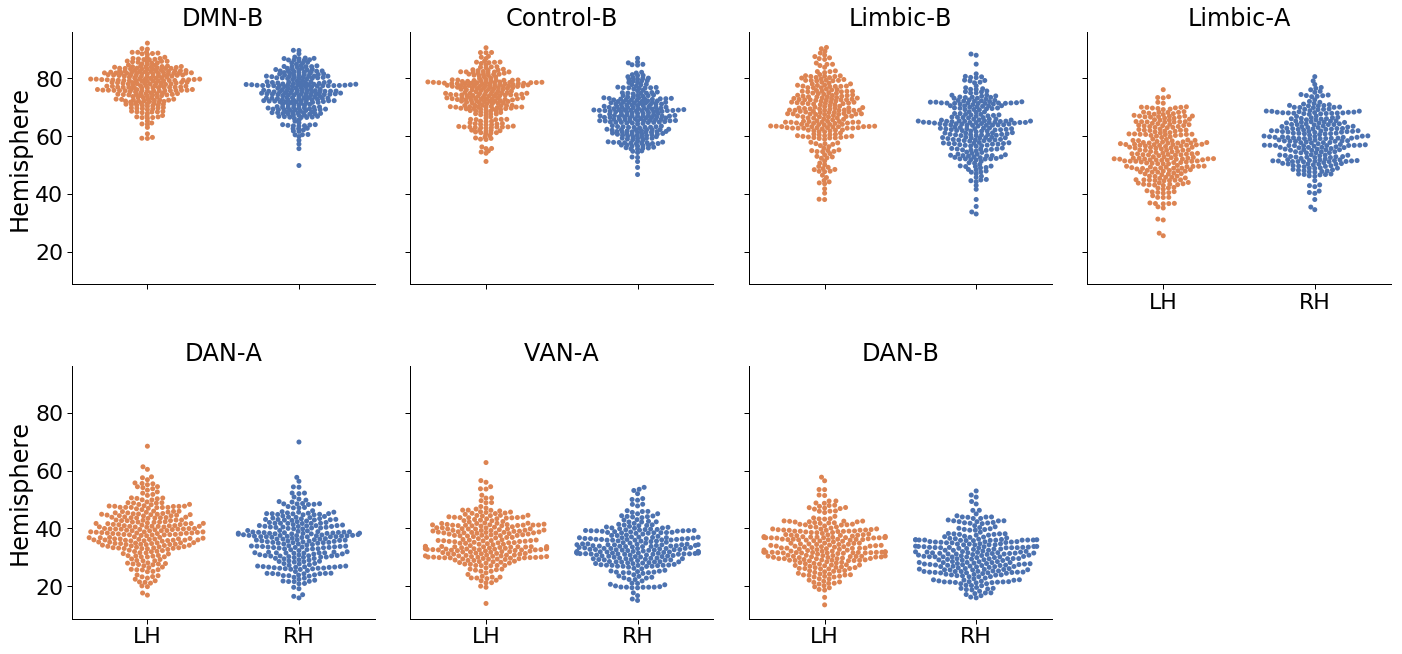


**Supplementary Figure 1.** Breakdown of gradient values for networks that show hemispheric differences in their position along the principal gradient.

**Supplementary Analysis of the Second Gradient**

**Results**

Our main analysis focussed on exploring hemispheric differences in the principal gradient. However, the gradient explaining the second largest amount of variance, which captures the separation between somatomotor and visual cortex, has shown functional associations in other studies (Margulies et al., 2016; McKeown et al., 2020). This prompted us to perform a supplementary exploratory analysis examining hemispheric differences in this second gradient, using the same methods as in our main analysis (excluding the analysis of individual differences). In this analysis, lower values represent proximity to the visual end, while higher values represent proximity to the somatomotor end.

The second gradient revealed a global mean difference: parcels in the left hemisphere were globally closer to the visual end, while parcels in the right hemisphere were closer to sensorimotor and auditory cortex (paired samples t-test: t(252) = 11.3, p < .001). Participants’ left and right hemisphere mean gradient values were very highly correlated (Pearson’s r = 0.95, p < .001), despite this global difference.


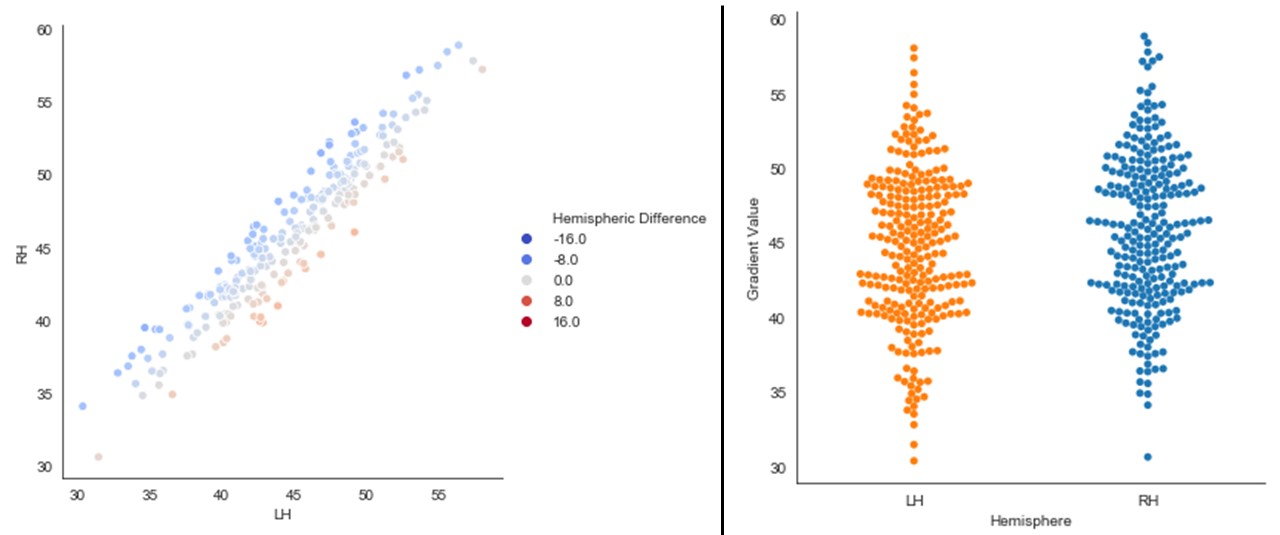


**Supplementary Figure 2.** The left panel depicts a linear relationship in our sample’s mean left and right hemisphere values in the second gradient. The right panel depicts the distributions of these mean global hemispheric values per participant in our sample. The ‘Hemispheric Difference’ legend of the scatterplot depicts the result of subtracting the L – R mean gradient loadings for the whole hemisphere per participant (negative values show right bias).

Given that our analysis pipeline involved a gradient alignment step, the gradient decomposition of our 253 participants closely replicated the second gradient reported by Margulies et al. (2016) and McKeown et al. (2020), at both the network and parcel level (see Supplementary Figure 3).


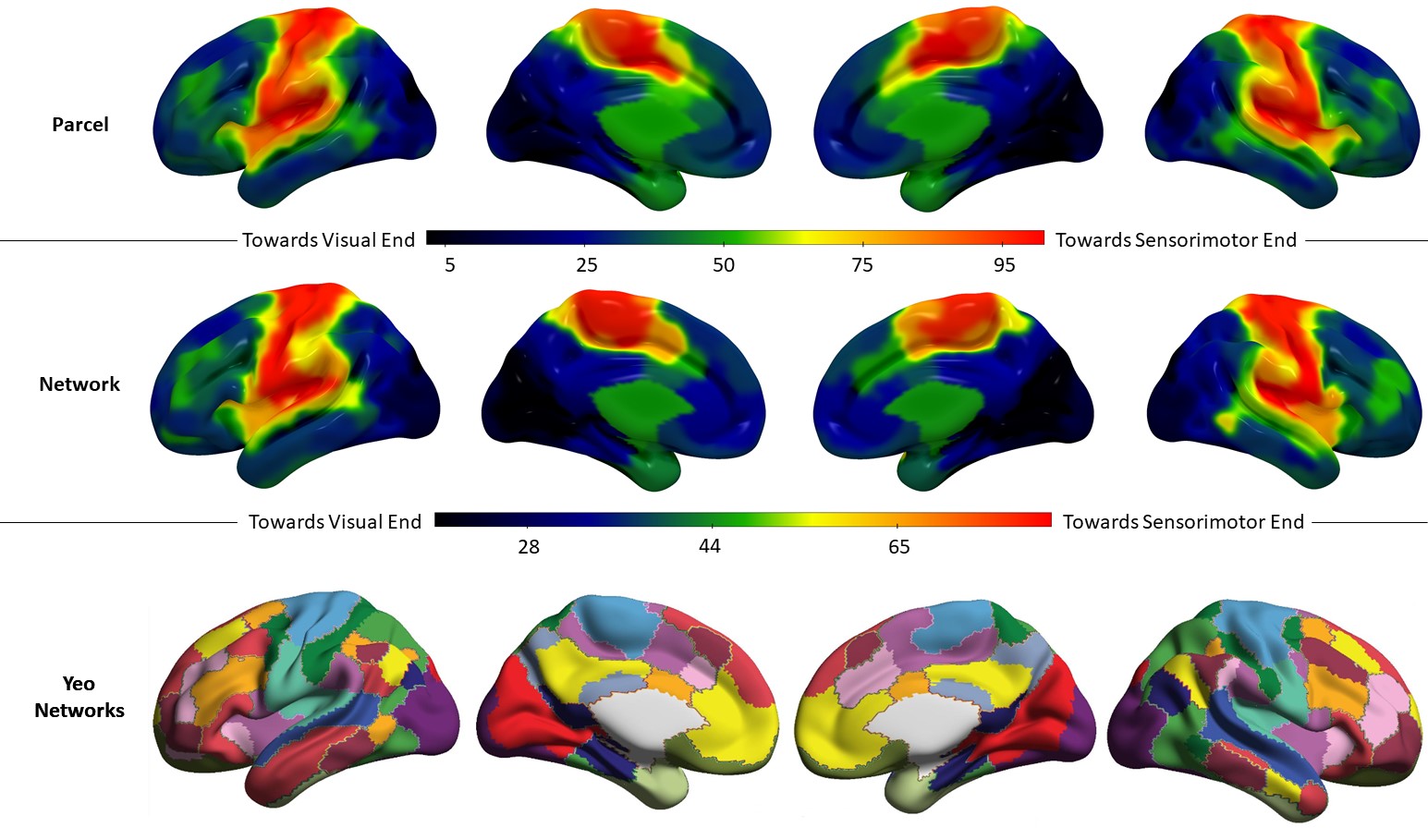


**Supplementary Figure 3.** Top row: Group mean second gradient value for each parcel in Schaeffer’s 400-parcel solution for our sample of 254 participants. Middle row: Group mean second gradient value for each network in Yeo’s 17-network solution for our sample of 254 participants. Gradient units are arbitrary and have been normalised on a 0-100 minmax scale. Bottom row: 17 network parcellation by Yeo et al. The colour code followed in this figure replicates that of Buckner et al. (2011)

Next, we averaged all parcels that fell within each of Yeo’s 17 networks in the left and right hemispheres separately, and then subtracted LH – RH, z-scoring the resulting differences. The results are shown in Supplementary Figure 4. The areas in warm colours are nearer the sensorimotor/auditory apex in the left hemisphere relative to the right, while the cool colours represent greater proximity to the visual apex in the left hemisphere compared to the right.


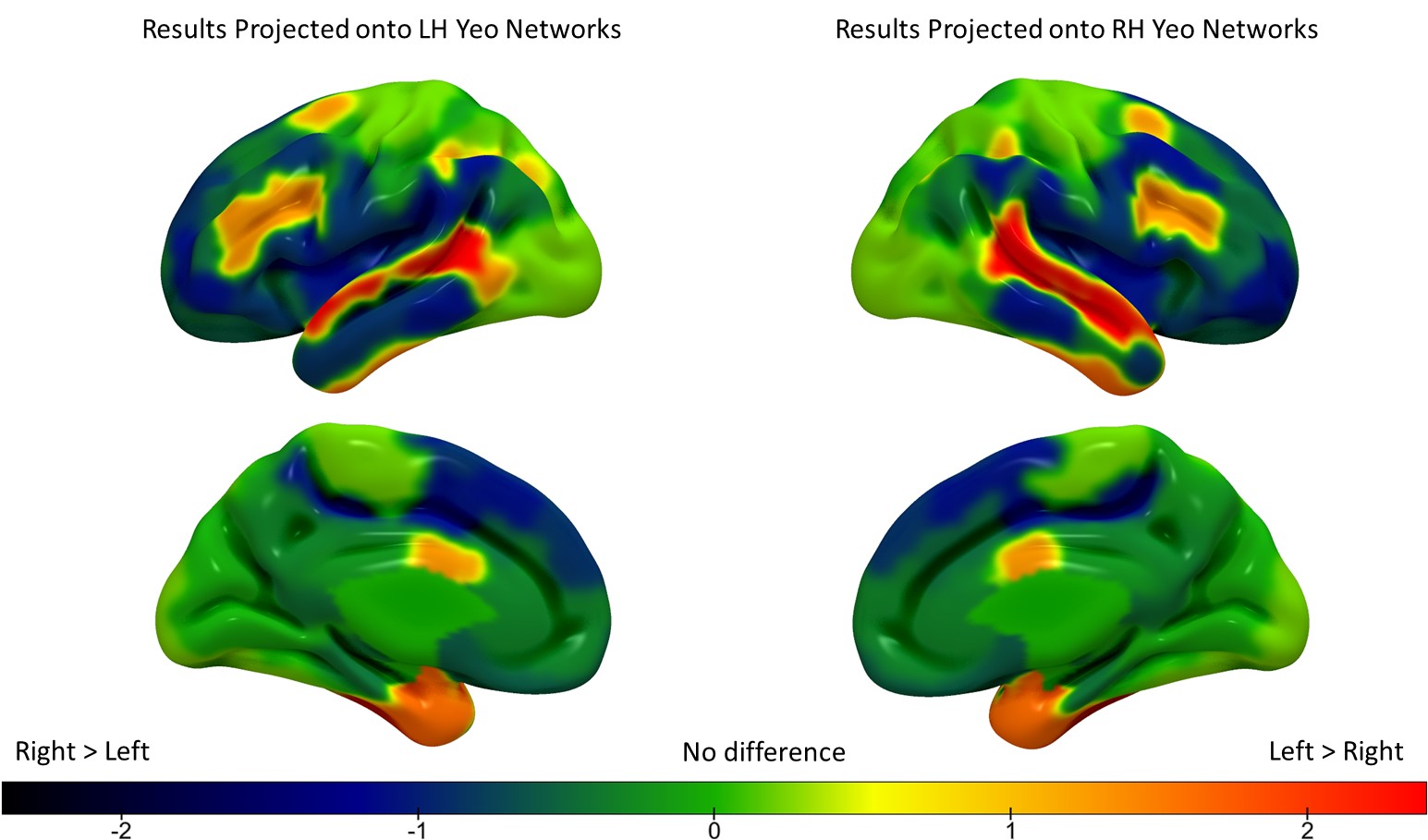


**Supplementary Figure 4.** Hemispheric differences in second gradient values across the 17 Yeo networks (z-scored). The warm colours represent a greater second gradient loading in the LH compared to RH, and the cool colours represent a greater second gradient loading in RH compared to LH.

We performed a repeated-measures ANOVA to formally test for differences in the second gradient loadings at the network level (2 hemispheres by 17 networks), controlling for global hemispheric differences in gradient values by entering each participant’s global LH – RH difference value as a covariate of no interest. The results of this ANOVA revealed significant main effects of hemisphere (F(1,251) = 769.12, p < .0001, ηp2 = .75), and network (F(8.37, 2101.24) = 1959.59, p < .0001, ηp2 = .89), as well as a significant hemisphere by network interaction (F(10.95, 2749.75) = 22.11, p < .0001, ηp2 = .08; all values with Greenhouse-Geisser correction to account for violations of the sphericity assumption). Subsequent post-hoc tests comparing the left and right hemispheres for each network (using permutation testing with 5,000 simulations to establish significance; Bonferroni-corrected for 17 comparisons) revealed that these hemispheric differences were robust for five networks: DMN-B, Control-B, Limbic-A, Control-A, and Temporoparietal (Supplementary Figure 5).


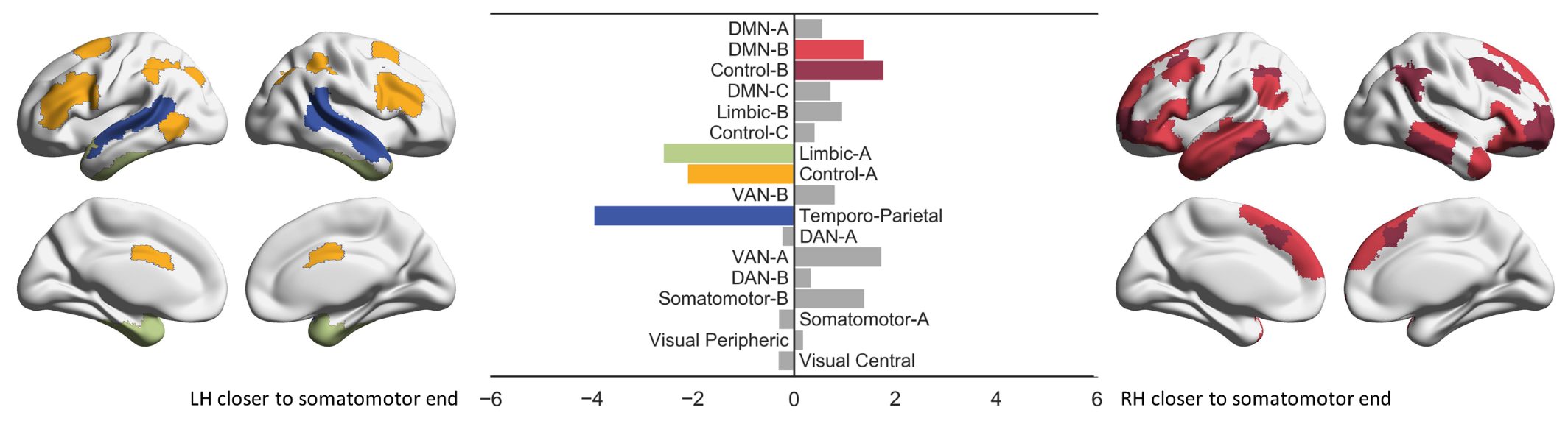


**Supplementary Figure 5.** Results of pairwise bootstrapped replicates permutation testing of LH vs RH means for each network (5,000 simulations, Bonferroni-corrected alpha for 17 comparisons equal to 0.00294). The size of each bar reflects the normalized (0-100) empirically observed mean difference across the hemispheres for each network. Coloured bars denote networks that showed significant differences and are colour-coded to the significant networks in the brain maps.

**Discussion**

Similar to the principal gradient, the finding of significant hemispheric differences in the Control-B and DMN-B networks in the second gradient is compatible with differential organisation of these networks in the left and right hemisphere. The greater proximity to the visual end in the left hemisphere relative to the right of these networks involved in semantic cognition might be related to the importance of vision in human semantic memory. Temporoparietal, Limbic-A and Control-A networks showed a greater proximity to the sensorimotor/auditory end of Gradient 2 in the left hemisphere; at least some of these regions are implicated in language functions that involve auditory-motor integration.

**Details of Gradient Extraction Process**

The supplementary materials reproduced in this section were originally published as part of McKeown et al. (2020) under a Creative Commons License ([CC BY-NC-ND](https://www.google.com/url?q=http://creativecommons.org/licenses/by-nc-nd/4.0&source=gmail-html&ust=1624348271017000&usg=AFQjCNG7DTKHlBzXgfwaHK4Qjogd6AI2vA))

**
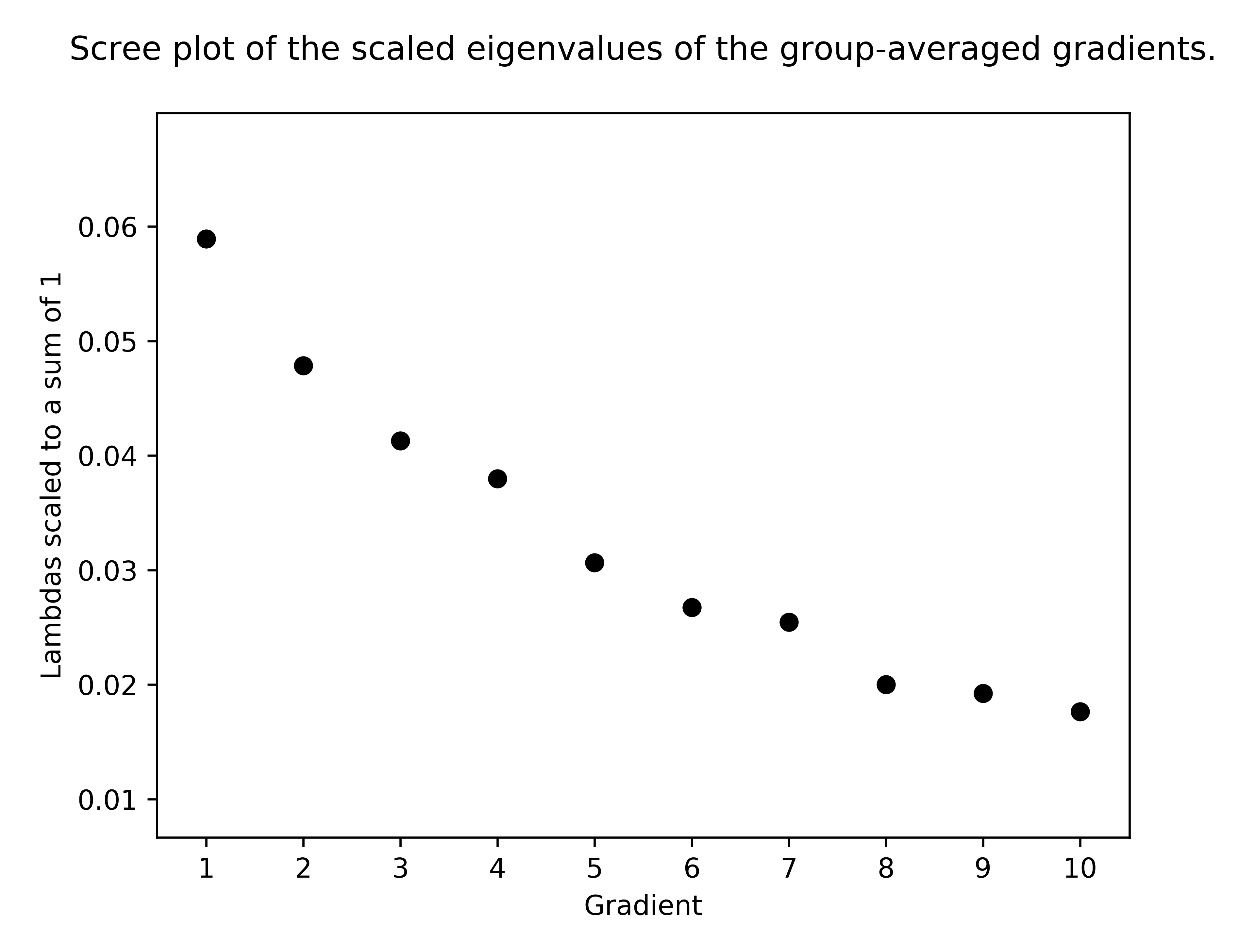
Supplementary Figure 6.** *Scree plot showing the proportion of variance explained by each of the group-averaged whole-brain connectivity gradients one to ten.* Y-axis shows the eigenvalues scaled to a sum of 1. X-axis shows the gradient number. The first three gradients were retained for further multivariate analyses as these gradients have the clearest mapping to cognitive function e.g. (Murphy et al., 2019, 2018; Turnbull et al., 2020).

**
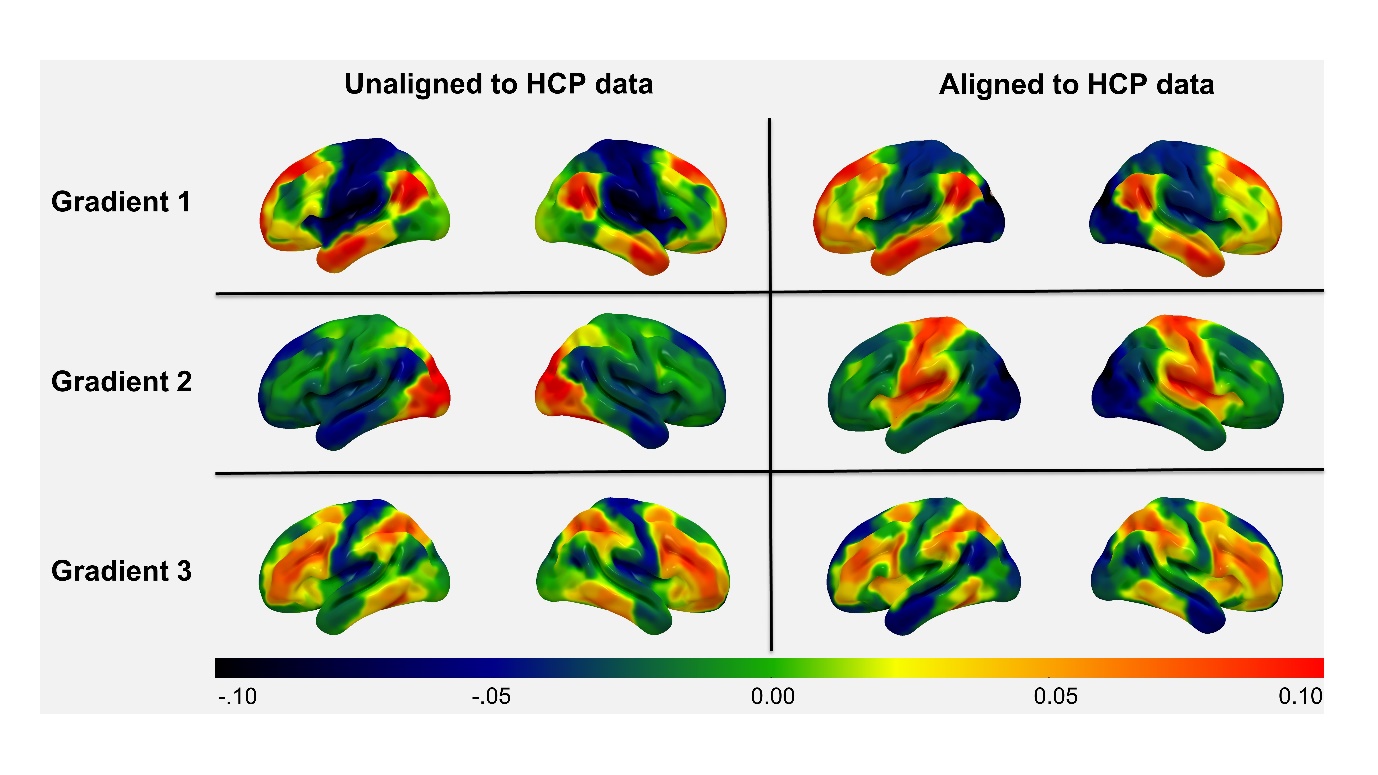
Supplementary Figure 7.** *Demonstration of how aligning the group-level gradients to a subsample of the HCP dataset using Procrustes rotation changes the first three group-level gradients.* Regions that share similar connectivity profiles fall together along each gradient (similar colours) and regions that have more distinct connectivity profiles fall further apart (different colours). It is important to note that the positive and negative loading is arbitrary and can flip each time the diffusion embedding is applied to the data. For example, in this figure, the visual cortex along gradient two has a positive loading in the unaligned map but has a negative loading in the aligned map. Thus, differences in loadings are not meaningful and occur randomly.


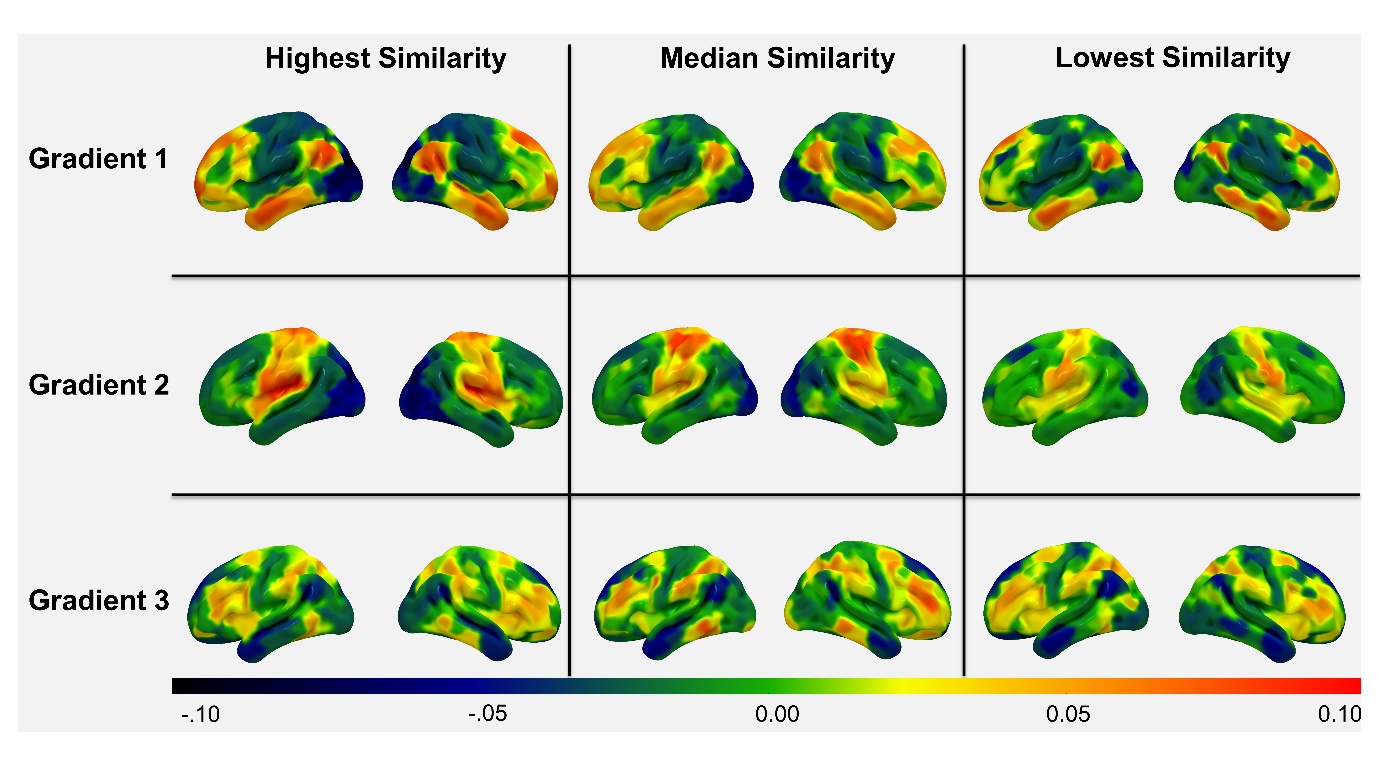


**Supplementary Figure 8.** *Individual-level connectivity gradients one to three which have the highest (left), median (middle) and lowest (right) similarity with the respective group-level gradients to demonstrate the variability of gradients across participants in the current sample*. Regions that share similar connectivity profiles fall together along each gradient (similar colours) and regions that have more distinct connectivity profiles fall further apart (different colours). The positive and negative loading is arbitrary.

| **Extracting 3 gradients:** | **Minimum** | **Maximum** | **Mean** | **Std. Deviation** |
| --- | --- | --- | --- | --- |
| Gradient 1 | 0.31 | 1.31 | 0.84 | 0.21 |
| Gradient 2 | 0.28 | 1.48 | 0.84 | 0.25 |
| Gradient 3 | -0.07 | 1.04 | 0.57 | 0.19 |
| **Extracting 10 gradients:** | **Minimum** | **Maximum** | **Mean** | **Std. Deviation** |
| Gradient 1 | 0.7 | 1.76 | 1.36 | 0.16 |
| Gradient 2 | 0.9 | 1.85 | 1.37 | 0.16 |
| Gradient 3 | 0.58 | 1.38 | 1.12 | 0.12 |

Table S2. This table shows the improvement in the degree of fit (or similarity) between individual-level and group-level gradients when extracting ten gradients compared to only extracting three gradients. Mean similarity was calculated by averaging all participant’s R-to-Z transformed Spearman Rank correlation coefficients for each respective gradient.

|  | **Aligned to HCP** | **Unaligned to HCP** |
| --- | --- | --- |
| Gradient 1 | 0.62 | 0.4 |
| Gradient 2 | -0.47 | 0.23 |
| Gradient 3 | -0.45 | -0.38 |
| Gradient 4 | -0.2 | 0.07 |
| Gradient 5 | -0.18 | -0.03 |

Table S3. Spearman rank correlation values for the first five aligned and unaligned group-level gradients with the first five group-level gradients reported in Margulies et al (2016). This demonstrates that aligning the group-level gradients to the subsample of HCP data improves correspondence between the gradients calculated in the current study and previous literature.

**Details of Behavioural Results**

|  | RJT Weak | RJT Strong | RJT Picture | RJT Word | Perceptual |
| --- | --- | --- | --- | --- | --- |
| RT (ms) | 1766.17 (196.2) | 1302.40 (162.41) | 1215.13 (255.64) | 1539.35 (187.36) | 1596.67 (239.67) |
| Accuracy (%) | 77.5 (7.6) | 95.59 (4.13) | 92.76 (4.61) | 89.55 (7.1) | 85.53 (10.3) |
| Efficiency | 2323.01 (454.6) | 1371.3 (205.03) | 1316.98 (286.98) | 1743.49 (312.64) | 1906.22 (437.55) |

Table S4. Descriptive statistics of the behavioural variables of each condition that comprise the Semantic Relatedness Judgement Task (RJT) and the perceptual (control) task in our sample at the group level. They are reported in the format M(SD), the time units are milliseconds and the accuracies are percentages.


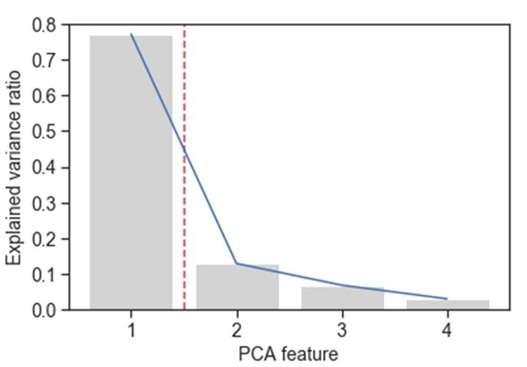


**Supplementary Figure 9.** Explained variance by PCA component of the dimensionality reduction performed on the scores of the four conditions of the semantic task of Study 1.

|  | Total Attempted | Total Correct | Accuracy: % correct over attempted | Accuracy: % correct over total |
| --- | --- | --- | --- | --- |
| M (SD) | 28.83 (6.9) | 19.06 (6.13) | 66.76 (16.8) | 58.79 (13.92) |

Table S5. Descriptive statistics of participants’ performance in the Raven’s Advanced Progressive Matrices task in our sample at the group level.

The Digit Span task group mean for number of items remembered was 6.8, with a standard deviation of 1.4.

**Study 2**

**Details of Behavioural Results**

|  | **Difficulty** | | | | |
| --- | --- | --- | --- | --- | --- |
| **Condition** | 1 | 2 | 3 | 4 | 5 |
| Semantic Related | 1.01 (0.37) | 1.03 (0.35) | 1.12 (0.37) | 1.19 (0.34) | 1.21 (0.34) |
| Semantic Unrelated | 1.14 (0.35) | 1.12 (0.32) | 1.15 (0.34) | 1.21 (0.33) | 1.27 (0.35) |
| Working Memory Correct | 0.88 (0.15) | 0.93 (0.19) | 0.98 (0.22) | 0.99 (0.19) | 0.96 (0.18) |
| Working Memory Incorrect | 1.08 (0.40) | 1.09 (0.27) | 1.07 (0.32) | 1.12 (0.28) | 1.10 (0.29) |

Table S6. Descriptive statistics of participants’ reaction time (RT) in the parametric effects analysis tasks (Semantic Relatedness and Working Memory) across the five difficulty levels.


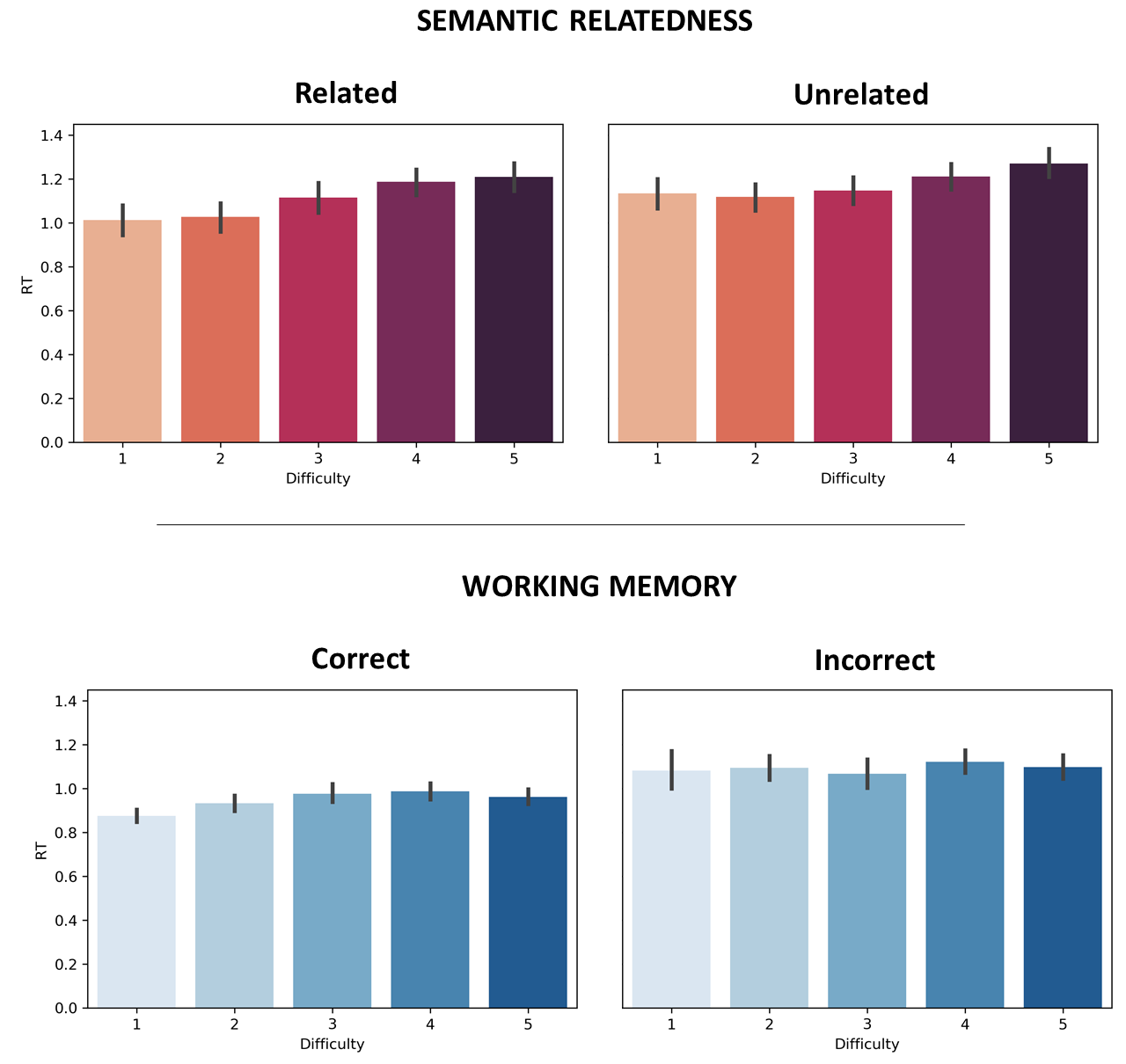


**Supplementary Figure 10.** Behavioural results in the parametric effects analysis tasks of Study 2 (Semantic Relatedness and Working Memory). Top panel: RT for semantic decisions across 5 levels of word2vec for word pairs judged to be related and unrelated. Bottom panel: RT for working memory trials across 5 levels of load, for correct and incorrect decisions.

**Supplementary Analysis: Assumption Check for Efficiency Scores**

The validity of efficiency scores requires inspecting the data to make sure that assumptions are met. Bruyer and Brysbaert (2011) suggest that when analyses are limited to conditions with accuracy of 90%, there is an equivalent percentage of variance explained for RT and IES. Both Bruyer and Brysbaert (2011), and Vandierendonck (2017) agree that efficiency scores should only be used in cases where there is a positive correlation between RT and Proportion of Errors (PE). Vandierendonck (2017) adds that the efficiency scores should only be considered when both RT and PE show effects in the same direction (in cases where there are effects in both). We analysed our data to test these assumptions, and the results comply to a reasonable degree with two of them.

We inspected the accuracy of each condition in our semantic task, and all but one of them exceed 90% (the ‘Weak’ condition, with accuracy of 77%, with the average across the four being 89%; see Table S7). All contrasts of interest showed significant differences in both, RT and PE across conditions, and these effects were always in the same direction (i.e. mean RT for the weak condition was significantly greater than for the strong condition, and PE showed the same pattern; Table S8). On the other hand, all conditions showed significant negative correlations between RT and PE, with the exception of the Picture condition, which showed a near-zero, non-significant correlation (Table S7).

In any case, following Bruyer’s reasoning, the unjustified use of efficiency scores would make the data noisier, reducing the percentage of variance explained and obscuring smaller effects. This would not affect the results we found (but would increase Type 2 errors). Likewise, Vandierendonck’s study concluded that the efficiency score is a valid measure with desirable statistical properties (yields symmetrical distribution, captures RT and PE in a balanced fashion). Future studies could improve the analyses using more sophisticated integrative metrics, such as LISAS, to maximise the variance explained, and potentially find smaller effects (Vandierendonck, 2017).

|  | RJT Weak | RJT Strong | RJT Picture | RJT Word |
| --- | --- | --- | --- | --- |
| Average Accuracy | 78% | 96% | 93% | 90% |
| Correlation of Reaction Time and Proportion of Errors (r, p) | r=-0.38  p<.001 | r=-0.23  p<.001 | r=0.02  p=.84 | r=-0.35  p<.001 |

Table S7. Average accuracy and correlation between reaction time and proportion of errors per condition of the semantic task.

|  | Weak > Strong | Word > Picture |
| --- | --- | --- |
| RT | T=55.35* | T=17.71* |
| Accuracy | T=33.27* | T=6.21* |

Table S8. Paired t-test values depicting the significance and direction of effects of interest in the semantic task. Note: * = p<.0001
